# Montmorillonite-supported Ag/Sn@CeO_2_*via* phyto-mechanochemical engineering: enhanced crystal violet adsorption and CO_2_ capture

**DOI:** 10.1039/d6ra07930j

**Published:** 2026-10-19

**Authors:** Mobeen Ur Rehman, Muhammad Usman, Muhammad Babar Taj, Amal Marzoqi Altowerqi, Khloud Al-Thubaiti, Merfat M. Alsabban, Alya Awad Alshahri, Ismat Bibi, Francis Verpoort, Rajeh Alotaibi

**Affiliations:** a Institute of Chemistry, The Islamia University Bahawalpur Bahawalpur Pakistan dr.taj@iub.edu.pk; b Department of Physics, University of Taibah Madinah Saudi Arabia; c Department of Chemistry, King Abdulaziz University Jeddah Saudi Arabia; d Department of Chemistry, College of Science, University of Jeddah Jeddah 21959 Saudi Arabia; e Jubail Induustrial College Jubail Saudi Arabia; f State Key Laboratory of Advanced Technology for Materials Synthesis and Processing, Wuhan University of Technology Wuhan 430070 China; g Department of Chemistry, College of Science, King Saud University Riyadh 11451 Saudi Arabia raalotaibi@ksu.edu.sa

## Abstract

Crystal Violet (CV) dye and CO_2_ emissions represent two major environmental threats that demand cost-effective and multifunctional remediation materials. In this study, a silver/tin co-doped cerium oxide nanocomposite supported on montmorillonite (Mt-Ag/Sn@CeO_2_) was synthesized *via* continuous phyto-mechanochemical twin screw extrusion using *Melothria scabra* leaf extract as a green reducing agent. The composite was characterized by using FTIR, XRD, SEM, EDS, zeta potential, N_2_ adsorption–desorption isotherm, BJH pore size analysis, and ICP-OES. The adsorption performance of Mt-Ag/Sn@CeO_2_ and Ag/Sn@CeO_2_ toward CV dye was systematically evaluated by optimizing pH, contact time, adsorbent dose, initial dye concentration, and temperature. For CV dye removal, Mt-Ag/Sn@CeO_2_ achieved 96.08% removal compared with 89.22% for Ag/Sn@CeO_2_ (10 mg L^−1^ dye, 20 mg dose, pH 9, 298 K, 180 min). The maximum adsorption capacities, estimated from the Langmuir model, were 36.50 mg g^−1^ and 28.77 mg g^−1^, respectively. Adsorption data were best described by the Freundlich isotherm alongside the pseudo-second-order kinetic model, consistent with chemisorption on a heterogeneous surface with multilayer dye uptake. Thermodynamic analysis confirmed that the adsorption process is exothermic and spontaneous (Δ*G*° < 0 at all studied temperatures). Furthermore, Mt-Ag/Sn@CeO_2_ demonstrated CO_2_ adsorption capacity of 3.92 mmol g^−1^ at 298 K and 900 mmHg, surpassing several reported clay-based adsorbents. These results confirmed that the mechanochemically engineered Mt-Ag/Sn@CeO_2_ composite is an effective dual-function adsorbent with potential for simultaneous water and air remediation.

## Abstract 2

Twin-screw extrusion enabled phyto-mechanochemical synthesis of Mt-Ag/Sn@CeO_2_ nanocomposites for crystal violet adsorption (36.50 mg g^−1^) and CO_2_ capture (*K* = 0.0039 mmol g^−1^ mm Hg^−1^ at 298 K).

## Graphical abstract

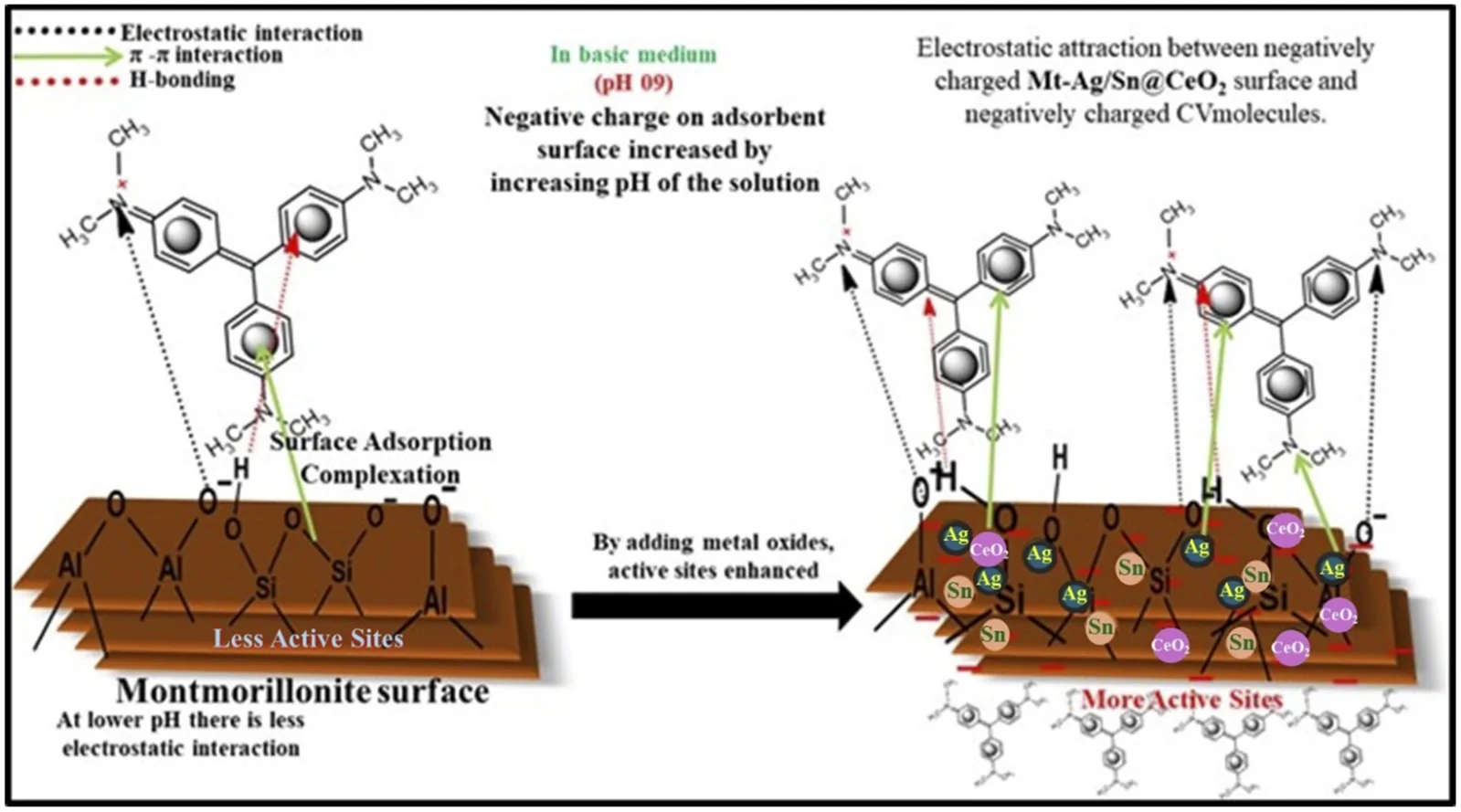

## 1. Introduction

In recent decades, due to population growth and industrial development, there has been a significant increase in global freshwater demand, leading to a corresponding rise in water usage.^1,2^ A large volume of inadequately treated effluents is continuously discharged into natural water bodies, introducing a wide range of toxic pollutants, including agricultural chemicals, organic compounds, and pH-altering substances.^3,4^ Industrial sectors such as paper manufacturing, petroleum refining, and pharmaceuticals are major contributors to this pollution, as their effluents deplete dissolved oxygen in water bodies and threaten aquatic life.^5^ Among these pollutants, synthetic dyes released from the textile industry represent one of the most hazardous categories, owing to their high visibility at low concentrations.^6^

Crystal violet is a cationic triphenylmethane dye. It is widely used in textile dyeing, paper printing, and biological staining. It is water-soluble with a highly conjugated aromatic structure and a cationic charge.^7^ The positive charge on the CV molecule allows it to bind tightly to negatively charged surfaces, making its removal from water particularly challenging. CV has been shown to cause mutagenic and carcinogenic effects in biological systems, even at trace levels.^8^ Conventional wastewater treatment techniques, such as flocculation, biological oxidation, and chlorination, fail to fully mineralize CV due to its structural stability. Recent advances in membrane and electrochemical separation technologies have further improved the removal of dissolved contaminants from water.^9–12^ A wide range of sustainable adsorbents has been developed for cationic dye removal, including biopolymer composites, waste-derived activated carbons, and functional polymers, with performance frequently optimized through statistical experimental design and theoretical modelling.^9,13–15^ Among the available alternatives, adsorption has emerged as the most reliable approach.^16^ It offers operational simplicity, high removal efficiency, and the ability to treat large volumes of contaminated water economically. Nanostructured metal oxide composites have attracted growing interest as adsorbents for cationic dyes owing to their tunable surface chemistry and high active site density.

Cerium oxide (CeO_2_) is a well-established metal oxide for environmental remediation. Its surface is rich in hydroxyl groups and oxygen vacancies, which serve as active binding sites for pollutant molecules. The incorporation of Ag into the CeO_2_ lattice has been shown to enhance surface electron density and improve adsorption of anionic and cationic species. Similarly, Sn doping increases the density of Lewis acid sites on the CeO_2_ surface, strengthening interactions with electron-rich pollutant molecules.^17^

Montmorillonite is an abundant natural clay mineral with a characteristic 2 : 1 layered silicate framework. Its high surface area and permanent negative layer charge make it one of the most effective natural adsorbents for cationic pollutants.^18^ Inorganic-modified montmorillonite (Mt) exhibits potentially useful structural incorporation, flexibility, ion exchange capability, and surface precipitation, which allow for the capture of cationic and anionic dye contaminants.^19^ Beyond its role in water treatment, Mt has demonstrated relevance in CO_2_ management. Effective carbon management is central to climate-resilient strategies across both environmental and agricultural systems.^20^ In this context, solid sorbents capable of capturing CO_2_ from gas streams have attracted significant attention. Its swelling behavior under CO_2_ exposure allows the gas to intercalate between the clay layers. This CO_2_ uptake is further promoted by the interaction of gas molecules with interlayer cations.^21,22^ These properties, *i.e.*, strong dye adsorption and intercalation, make Mt an ideal structured support for designing a dual-function composite adsorbent.

Chemical synthesis of metal oxide nanoparticles conventionally depends on strong reducing agents, organic stabilizers, and energy-intensive processing conditions. These requirements generate hazardous waste and limit scalability for practical applications.^23^ Biogenic synthesis routes, which exploit the reducing and capping ability of plant-derived phytochemicals, offer a sustainable pathway to nanoparticle fabrication.

*Melothria scabra*, a climbing herb of the Cucurbitaceae family, produces leaves containing a diverse range of phenolic acids, flavonoids, and terpenoids. The leaves extract acts as a multifunctional bio-derived additive during twin-screw extrusion. The phytochemicals present in plant extracts, such as phenolic compounds, terpenoids, flavonoids, proteins, sugars, and other oxygen-containing biomolecules, have shown effective metal-reducing and particle-stabilizing activity in previous nanoparticle studies.^24^ Such biomolecules can ease electron transfer/precursor conversion, while their functional groups can adsorb onto particle surfaces and offer capping and stabilization, thereby limiting uncontrolled particle growth and aggregation. The extract may therefore contribute simultaneously to precursor activation, nucleation, and surface stabilization during the solvent-minimized extrusion process. It is consider that phenolic OH groups donate electrons to reduce the Ce^4+^, Ag^+^, and Sn^2+^ precursors, while the resulting carbonyl and hydroxyl functionalities coordinate to the nascent nanoparticle surfaces, restricting crystal growth and preventing aggregation. This dual reducing-capping role eliminates the need for separate synthetic surfactants and chemical reductants.^25^

Conventional routes^26^ for preparing clay/metal oxide composites including co-precipitation, sol–gel, and hydrothermal methods, typically requires large volumes of organic solvents, prolonged reaction times of 12–24 h, sequential washing and filtration steps, and batch wise operation that limits throughput.

Twin-screw extrusion differs fundamentally in that the reaction proceeds in the solid state under continuous mechanical shear.^27^ The mechanochemical reaction complete fastly as compared with several hours for solution-based routes and generates no filtrate waste stream. Because extrusion is a continuous rather than batch process, scale-up is achieved by extending operating time rather than by increasing vessel size, making the method directly transferable to industrial production. The comparative assessment of synthesis approaches and the present phyto-mechanochemical twin-screw extrusion method is given in Table S1 (SI). Critically, in the extrusion process the metal oxide nanoparticles nucleate *in situ* within the sheared clay matrix, rather than being pre-formed and mixed with the support as in conventional impregnation methods. This ensures strong interfacial contact between the clay lamellae and the growing nanoparticles, promoting uniform dispersion and limiting aggregation on the support surface.^28,29^

Although plant-mediated Ag/Mt nanocomposites^30^ and hydrothermally synthesized CeO_2_/Mt composites^31^ have previously been reported, the integration of plant-extract-mediated chemistry with twin-screw extrusion for the fabrication of an Ag/Sn-modified CeO_2_-montmorillonite architecture has not been adequately explored. Herein, twin-screw extrusion is employed as a high-shear reactive assembly platform that combines green chemical functionality with intensive solid-state mixing, providing a potentially solvent-minimized and scalable route to Mt-Ag/Sn@CeO_2_.

The synthesized composite was evaluated for two distinct environmental functions: the adsorptive removal of crystal violet CV dye and the capture of CO_2_ gas. The specific objectives of this study were: (i) to synthesize and characterize Mt-Ag/Sn@CeO_2_ and Ag/Sn@CeO_2_; (ii) to optimize CV adsorption parameters; (iii) to evaluate adsorption isotherms, kinetics, and thermodynamics; and (iv) to assess CO_2_ adsorption performance. This integrated approach, combining green mechanochemical synthesis, bimetallic Ag/Sn doping, and a clay-supported composite design, provides a single dual-function adsorbent for simultaneous water and air remediation.

## 2. Experimental

### 2.1. Materials

Montmorillonite used as a solid support was purchased from Daejung Co., Ltd Ceric ammonium nitrate, silver nitrate, Tin(ii) nitrate, sodium hydroxide, and ethanol were purchased from Merck and Sigma-Aldrich, all of which were of analytical grade.

### 2.2. Continuous-flow phyto-mechanochemical extrusion of Ag/Sn@CeO_2_ and Mt-Ag/Sn@CeO_2_

Solid reactants (0.01 mol of (NH_4_)_2_[Ce(NO_3_)_6_]; AgNO_3_, and Sn(NO_3_)_2_; each 3 mol% relative to Ce) were manually mixed with 10 mL of *Melothria scabra* leaf extract (extract preparation is given in the SI), serving as the reducing and stabilizing agent, for 5 minutes. The mixture was fed into the extruder (Thermo Fisher Process 11 Parallel Co-Rotating twin screw extruder, with an L/D ratio of 25) at 150 °C and at a speed of 100 rpm for 10 minutes. The resulting mixture was dried in the oven at 80 °C for 3 hours and calcined at 500 °C for 4 hours to yield the final product labeled as Ag/Sn@CeO_2_.

For the synthesis of montmorillonite fabricated Ag/Sn co-doped CeO_2_ (Mt-Ag/Sn@CeO_2_), 0.05 g of hydrated montmorillonite clay (soaked in 2.5 mL of distilled water to promote cation hydration and lamellae expansion) was added to the solid reactants in the above method, and the whole process was repeated. (The details of twin-screw extrusion parameters for reproducible synthesis are given in Table S2; SI).

### 2.3. Adsorptive removal of CV dye from water

A series of adsorption tests was conducted to investigate the adsorption capacity of Ag/Sn@CeO_2_ and Mt-Ag/Sn@CeO_2_ composites. These tests focused on evaluating the different operational factors on the adsorptive removal of CV dye from an aqueous system. In each test, 20 mg of adsorbent was added to 10 mL of 10 mg L^−1^ dye solution. The pH of the solution was adjusted to an alkaline value of 9 using 1 M NaOH, except in the pH dependence study described below, where pH was systematically varied. The solution was stirred continuously at 150 rpm (constant parameter) at room temperature using a magnetic stirrer. After every 20 minutes, a sample was taken from the solution, and absorbance was measured at the wavelength of 590 nm (*λ*_max_ of CV). To study the effect of different parameters on the adsorption of CV from solution, the experimental parameters, *e.g.*, initial dye concentration, contact time, pH, adsorbent dose, and temperature, were changed systematically. The solution pH was regulated using 1 M NaOH and 1 M HCl solutions, and readings were taken with a calibrated pH meter. The effect of temperature was explored over the range of 298–328 K. For quantitative analysis, a calibration curve was constructed using five CV standard solutions over the concentration range of 5–50 mg L^−1^. Absorbance was measured at *λ*_max_ = 590 nm and varied linearly with concentration, giving the regression equation *A* = 0.0180*C* − 0.0012 with a correlation coefficient of *R*^2^ = 0.999 Fig. S2. The % removal of CV dye and adsorption capacity (*q*_e_) were calculated by eqn (1) and (2), respectively.1
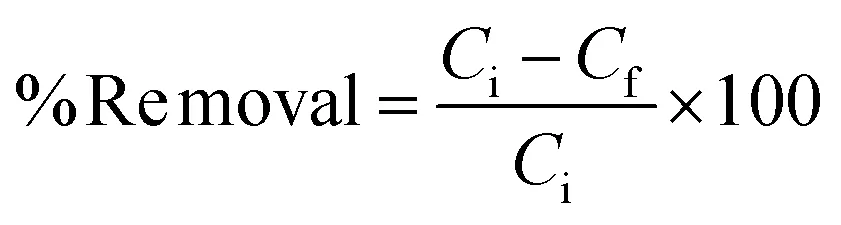


The *C*_i_ and *C*_f_ denote the initial and final concentrations of the dye.

2 *Q*_e_ = (*C*_0_ − *C*_e_)*V*/*W*

*C*_0_ and *C*_e_ (mg L^−1^) are the initial and equilibrium dye concentrations, *V* is the volume of solution (L), and *W* is the mass of the adsorbent (g). All adsorption experiments were performed in triplicate, and the results are expressed as mean values ± standard deviation.

### 2.4. CO_2_ adsorption study

CO_2_ adsorption isotherms were measured at 298 and 313 K using a Micromeritics ASAP 2020 volumetric adsorption analyzer. The adsorbent used was the Mt-Ag/Sn@CeO_2_ composite, selected based on its performance in prior dye adsorption studies. Approximately 500 mg of the composite was loaded into the analyzer's sample tube. Before measurement, the sample was degassed *in situ* to remove physisorbed moisture and gases. This was achieved by heating at 110 °C for 30 minutes under a continuous flow of pure N_2_, followed by 40 minutes under dynamic vacuum. Following activation, the sample tube was submerged in a temperature-controlled Micromeritics water bath, connected to a circulating bath to maintain precise isothermal conditions (298 and 313 K). High-purity CO_2_ (99.99%) was used as the adsorbate. The system introduced incremental doses of CO_2_ into the sample cell, allowing the pressure to equilibrate after each dose. The equilibration interval for each pressure point was set to 45 seconds. Adsorption isotherms were collected across a pressure range of 0 to 922 mmHg (123 kPa). The quantity of CO_2_ adsorbed at each equilibrium pressure was calculated by the system's software using the volumetric (manometric) method. Fig. 1 illustrates the CO_2_ adsorption setup.

**Fig. 1:**
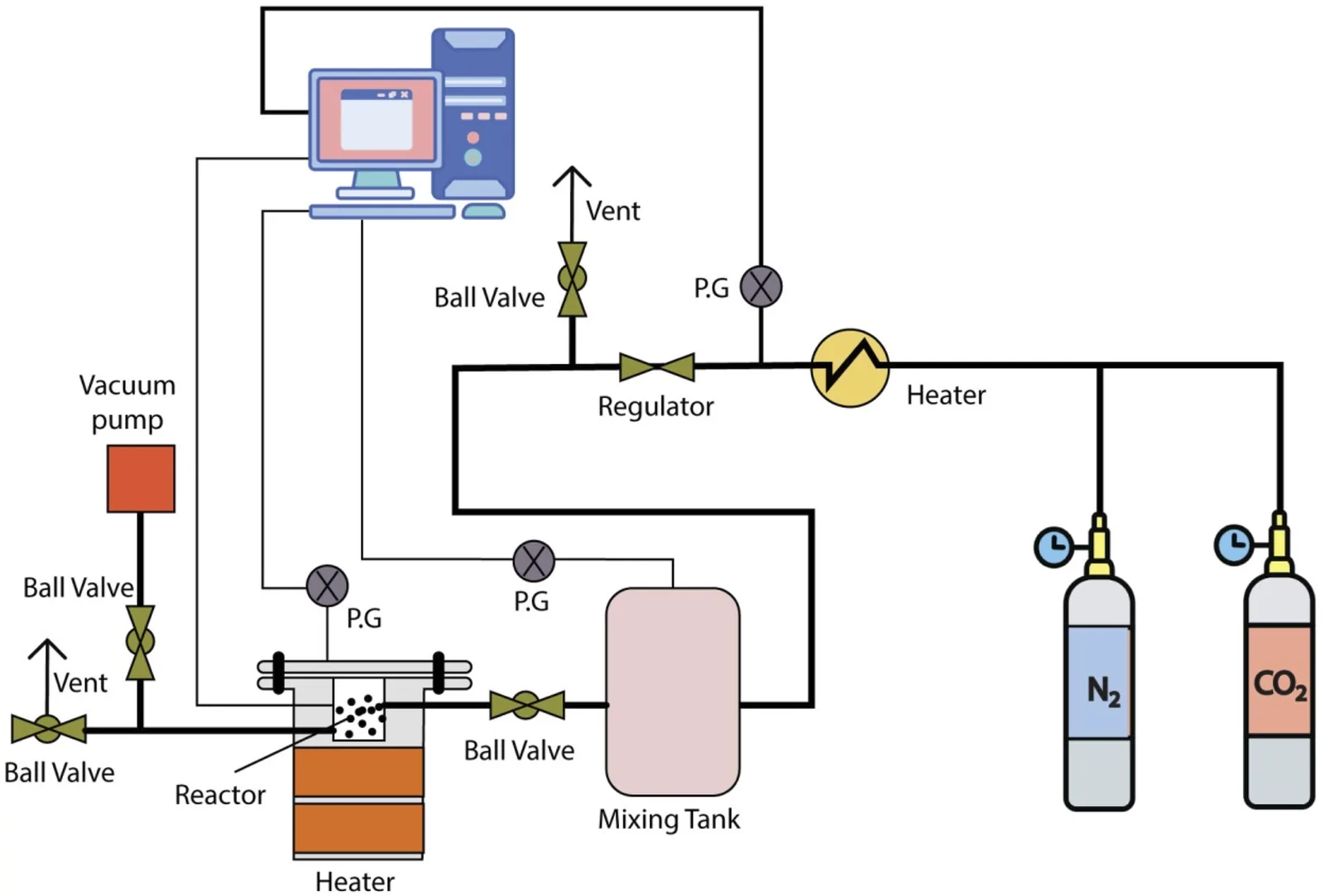
Schematic representation of the CO_2_ adsorption setup.

### 2.5. Characterizations

The functional groups of the synthesized Ag/Sn@CeO_2_ and Mt-Ag/Sn@CeO_2_ composites were analyzed using a BRUKER Tensor 27 FTIR spectrometer within the 4000–500 cm^−1^ range, employing KBr as the diluent. The surface morphological properties were scanned by the Nova NanoSEM 450 scanning electron microscope. The crystalline nature of the synthesized composites was determined using a JEOL X-ray diffractometer (model JDX-3532) with Cu Kα radiation. Nitrogen adsorption at −196 °C was conducted using a Quanta Instruments NOVA 1200, a specialized pore size analyzer. Before adsorption, the sample underwent a pretreatment at 200 °C for 17 hours.

## 3. Results and discussion

### 3.1. UV-visible analysis

The UV-visible absorbance spectrum of *Melothria scabra* and Mt-Ag/Sn@CeO_2_ composite is presented in Fig. S1. The *Melothria scabra* extract exhibits characteristic peaks at approximately 284 nm and 408 nm, corresponding to the presence of bioactive constituents such as phenolic compounds and flavonoids. These compounds serve as reducing and capping agents, facilitating the transformation of metal ions into their respective nanoparticles and stabilizing the resulting composite. The band at 284 nm is assigned to π to π* transitions of aromatic rings in phenolic compounds, while the band at 408 nm corresponds to transitions associated with the conjugated carbonyl systems of flavonoids. During extrusion, these phenolic hydroxyls donate electrons to reduce Ce^4+^, Ag^+^, and Sn^2+^, initiating nucleation, while the oxidized phytochemicals and remaining carbonyl groups chelate the nascent particle surfaces to restrict growth and prevent agglomeration. The colour change of the extract from green to brownish-yellow during preparation and the emergence of a new band at 469 nm in the composite spectrum are consistent with this bioreduction and capping process.^25^ The Mt-Ag/Sn@CeO_2_ composite exhibits a distinct peak at around 469 nm. This peak is indicative of the combined absorbance of the Ag/Sn@CeO_2_ composite.

### 3.2. Functional group analysis

The FTIR spectrum (Fig. 2a) of Mt-Ag/Sn@CeO_2_ exhibits a broad band in the range of 3460–3410 cm^−1^, corresponding to O–H stretching of surface hydroxyl groups and adsorbed water, in accordance with clay minerals and biosynthesized materials.^32,33^ The absorption near 1635 cm^−1^ represents H–O–H bending and residual organic/capping groups associated with green synthesis routes.^34^ A prominent Si–O stretching vibration of montmorillonite is recorded at 1074 cm^−1^ (ref. 32 and 33) while the peak around 823 cm^−1^ arises from Al–OH/Si–O lattice bending modes in the case of the Ag/Sn@CeO_2_ spectrum (Fig. 2b). The low-wavenumber region confirms the metal–oxide framework: Ce–O lattice vibrations of CeO_2_ occur at 490–510 cm^−1^ (ref. 35 and 36) while Sn–O/O–Sn–O stretching modes characteristic of Sn appear in the 560–650 cm^−1^ range.^37^ The wavenumber regions are less visible in the spectrum of Mt-Ag/Sn@CeO_2_, indicating the successful incorporation of montmorillonite. Metallic Ag does not exhibit intrinsic IR-active modes; however, its presence is inferred from shifts in hydroxyl and carboxylate bands due to biomolecule – Ag interactions.^37^

**Fig. 2:**
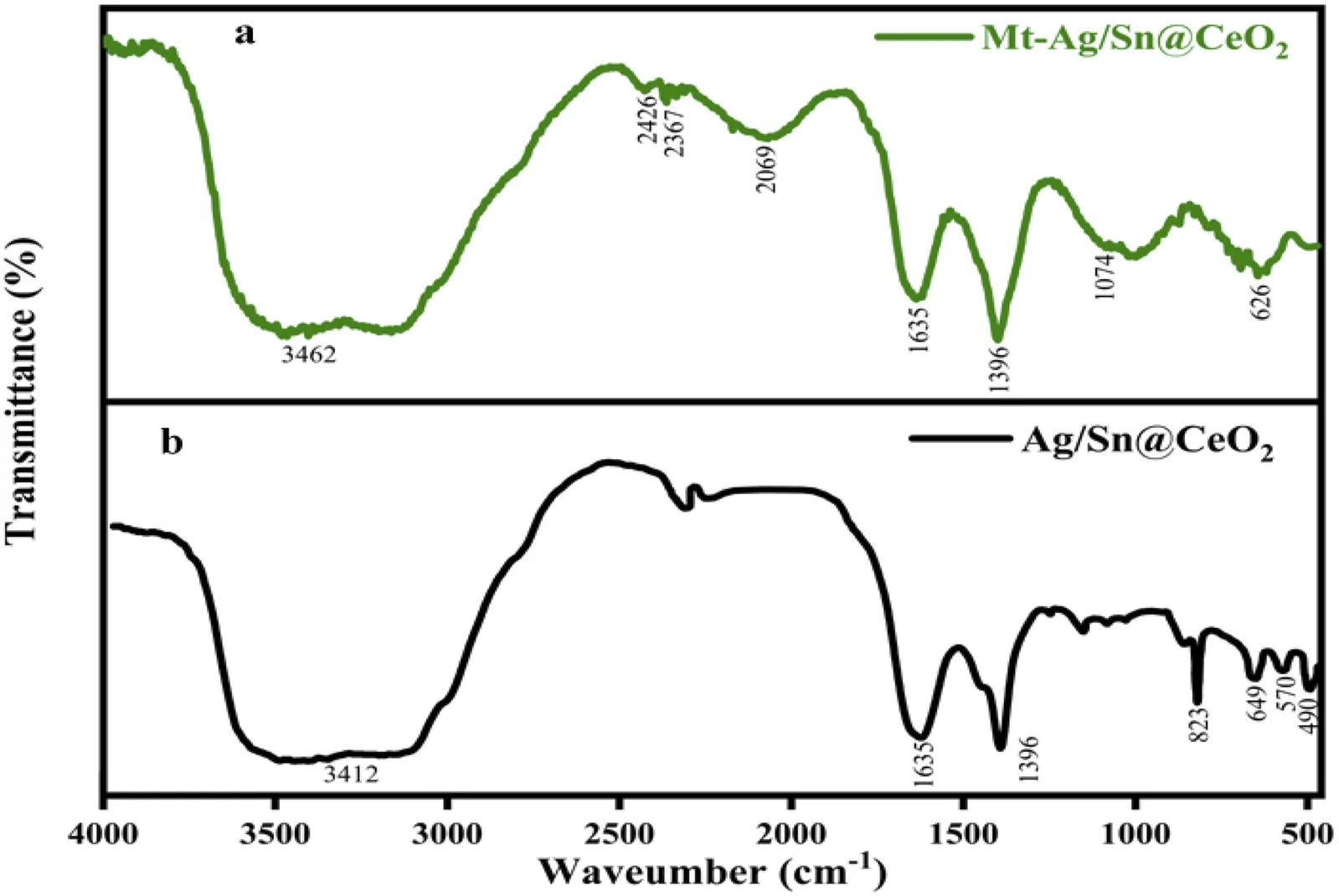
FTIR spectra of (a) Mt-Ag/Sn@CeO_2_ and (b) Ag/Sn@CeO_2_ composites.

### 3.3. XRD analysis

The structural features of Mt-Ag/Sn@CeO_2_ and Ag/Sn@CeO_2_ composites were examined through X-ray diffraction analysis, as presented in Fig. 3. The obtained diffraction patterns confirmed sharp and intense peaks, indicating the formation of well-crystallized metal oxide phases. Characteristic reflections of montmorillonite appeared at 2*θ* values of 6.1°, 18.9°, and 33.8°, corresponding to the (001), (020), and (110) planes (JCPDS card No. 82-1873). Distinct peaks observed at 28.3°, 32.5°, 47.1°, 56.8°, and 59.2° were indexed to the (111), (200), (220), (311), and (222) planes of CeO_2_ (JCPDS card No. 34-0394). Additional reflections at 38.3° (111), 42.9° (200), and 64.9° (220) were attributed to Ag (JCPDS card No. 89-3722), while peaks at 29.5° (200), 30.5° (101), 45.1° (211), 55.3° (301), and 62.1° (112) corresponded to Sn (JCPDS card No. 00-004-0673). These diffraction features collectively demonstrate the formation of Mt-Ag/Sn@CeO_2_, indicating the efficiency of *Melothria scabra* leaf extract in facilitating the bioreduction of metal precursors. The mean crystallite sizes were estimated from the Debye–Scherrer eqn (3).

3 Crystalline size (*D*) = *Kλ*/*β* cos *θ*

where *D* is the average crystallite size (nm), *K* is the Scherrer constant (0.9), *λ* is the X-ray wavelength (0.15406 nm for Cu Kα), *β* is the full width at half maximum of the diffraction peak (radians), and *θ* is the Bragg angle (degrees). The calculated mean crystallite size for Ag/Sn@CeO_2_ is 40.76 nm, and for Mt-Ag/Sn@CeO_2_ is 53.16 nm.

**Fig. 3:**
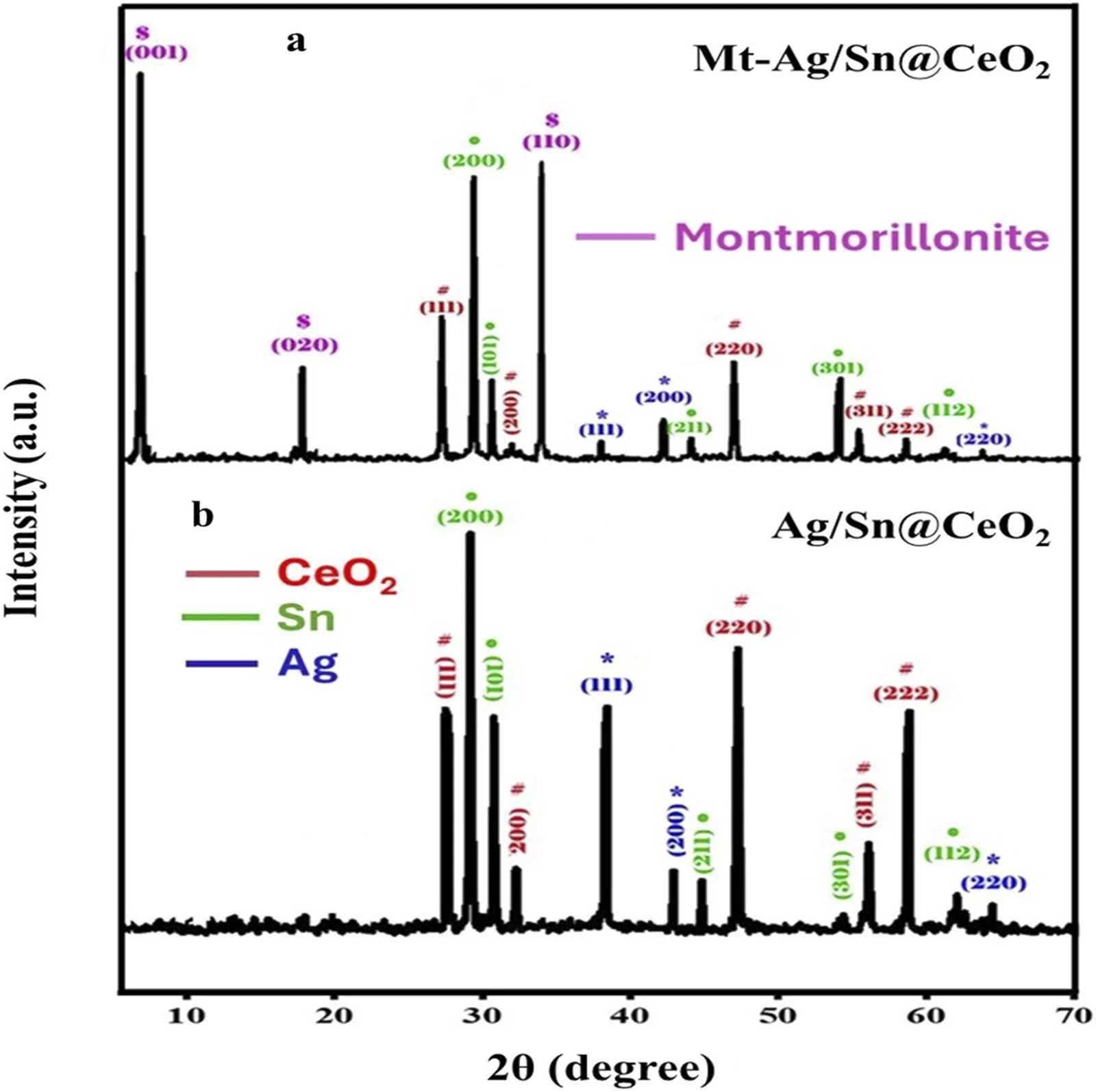
X-ray diffraction patterns of (a) Mt-Ag/Sn@CeO_2_ and (b) Ag/Sn@CeO_2_ composite.

### 3.4. Morphological analysis

The surface morphology of the Mt-Ag/Sn@CeO_2_ composite was evaluated using SEM analysis (Fig. 4a and b). The SEM image clearly reveals the sheet-like structure of montmorillonite, decorated with dispersed Ag/Sn co-doped metal–metal oxide particles of various morphologies. The sheet-like structure with uneven distribution of the final composite likely contributes to its high surface area, which significantly enhances its ability to adsorb pollutants.^38^ The EDS spectrum exhibited well-defined peaks of Ce, O, Si, and Al, which are derived from the CeO_2_ phase and Mt layers, whereas the clear signals of Ag and Sn confirm their composition. Homogeneous distribution of elements suggests that the metals are securely anchored, implying the stability of the composite for catalytic and adsorption purposes. The EDS spectrum (Fig. 4c) of Mt-Ag/Sn@CeO_2_ confirms that the Ag/Sn co-doped CeO_2_ particles are well anchored within the montmorillonite matrix.

**Fig. 4:**
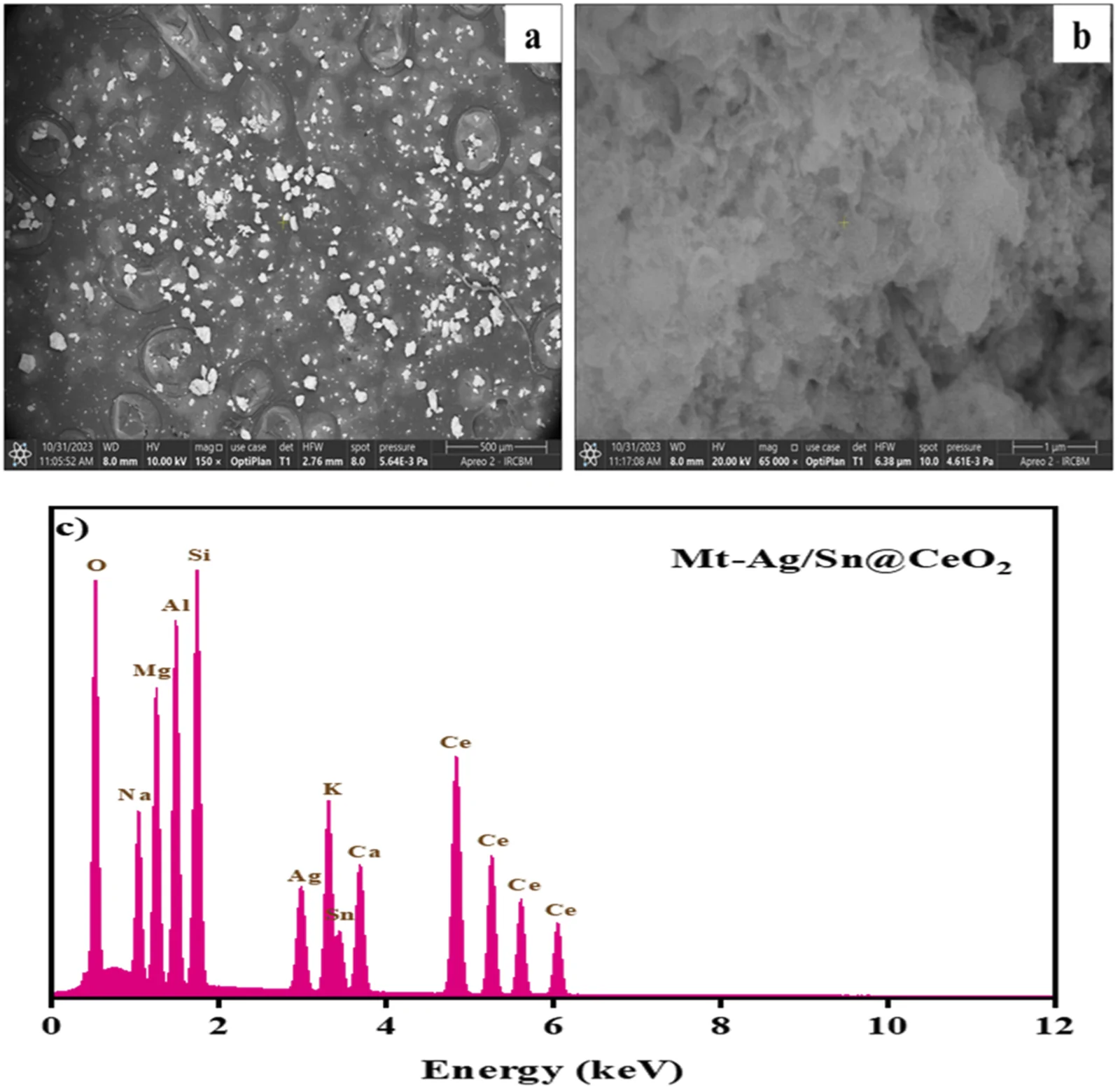
SEM micrographs of Mt-Ag/Sn@CeO_2_ at (a) 500 µm and (b) 1 µm scale bars, and (c) EDS spectrum of Mt-Ag/Sn@CeO_2_ composite.

### 3.5. Zeta potential and point of zero charge (PZC)

The composite Ag/Sn@CeO_2_ shows a zeta potential peak at −11.7 mV, indicating relatively poor stability with a higher tendency for aggregation, as compared to Mt-Ag/Sn@CeO_2,_ exhibiting a peak at −15.08 mV in Fig. 5a, indicating moderate colloidal stability with enhanced electrostatic repulsion and reduced aggregation tendency.^39,40^ The zeta potential of the Mt-Ag/Sn@CeO_2_ composite provides an approximation of surface charge density, electrostatic attraction, and the tendency to form aggregates. Strong electrostatic attraction is favored toward cationic CV when the surface is negatively charged. On the other hand, in an acidic environment (pH < PZC), the surface gains a positive charge, which suppresses electrostatic attraction and limits adsorption primarily to weaker non-covalent interactions. This indicates that the Mt-Ag/Sn@CeO_2_ composite will exhibit maximum CV adsorption at pH values above its PZC.^41^

**Fig. 5:**
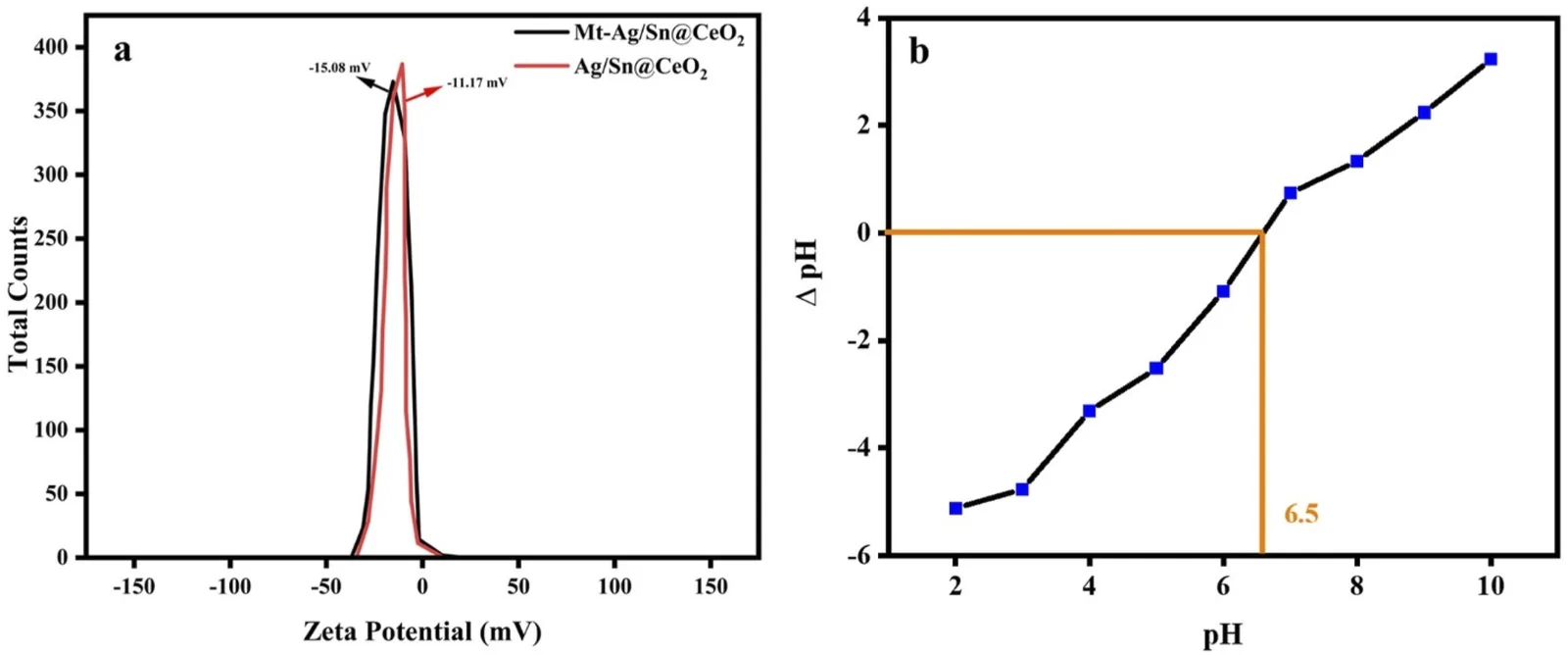
(a) Zeta potential distribution of Mt-Ag/Sn@CeO_2_ and Ag/Sn@CeO_2_ composite, (b) PZC graph of Mt-Ag/Sn@CeO_2_ composite.

It was noted that at pH 6.5, the adsorbent surface was electrically neutral (point of zero charge (PZC) as shown in Fig. 5b. Above pH 6.5, the surface takes on a negative charge, thus increasing the electrostatic attraction for cationic CV. In contrast, below the PZC, the surface is positively charged, resulting in repulsion and reduced uptake. The relationship between charge and pH is consistent with earlier studies on CV adsorption onto montmorillonite-based adsorbents, where the highest removal is also shown to occur at alkaline pH due to stronger electrostatic interaction.^42,43^

### 3.6. N_2_ adsorption–desorption isotherm and pore size

The analysis of specific surface areas by N_2_ adsorption–desorption showed BET surface area values of 67.48 m^2^ g^−1^ and 48.19 m^2^ g^−1^ for the Mt-Ag/Sn@CeO_2_ and Ag/Sn@CeO_2_ composites, respectively (Fig. 6a and c). The surface area increase with montmorillonite is likely attributed to montmorillonite's layered morphology, which provides more adsorption sites and reduces NPs agglomeration. Montmorillonite without additives typically has surface areas of 60 to 140 m^2^ g^−1^, depending on the interlayer cation and preparation conditions.^44^ A slightly reduced BET surface area was observed for the Mt-based composite compared to the unmodified Mt, which can be attributed to the partial obstruction of fine pores following the incorporation of Ag, Sn, and Ce oxides. Similar results have been shown from prior research, as surface area decreased upon immobilizing nanoparticles on Mt surfaces for a variety of clay-metal oxide hybrid materials.^45^ The N_2_ adsorption–desorption isotherm curves for both synthesized composites display Type IV isotherms with H3 hysteresis loop features. Such isotherm curves are seen in mesoporous materials with slit-shaped pores, a common characteristic of layered silicates, including montmorillonite.^46^ The pore size distribution graph further demonstrates the heterogeneous pore structure with mesoporosity for both materials, reflecting the multiple distinct peaks. The average pore diameter was 11.86 nm for Mt-Ag/Sn@CeO_2_, while it was 10.89 nm for Ag/Sn@CeO_2_ (Fig. 6b and d). This diameter confirms their classification within the mesoporous domain. The observed pore diameter distribution, characterized by peaks aligning with a Type IV isotherm, is indicative of mesoporous materials. The accompanying hysteresis loop, similar to type H3, suggests the presence of slit-shaped pores, a common feature in layered materials such as montmorillonite.^47,48^

**Fig. 6:**
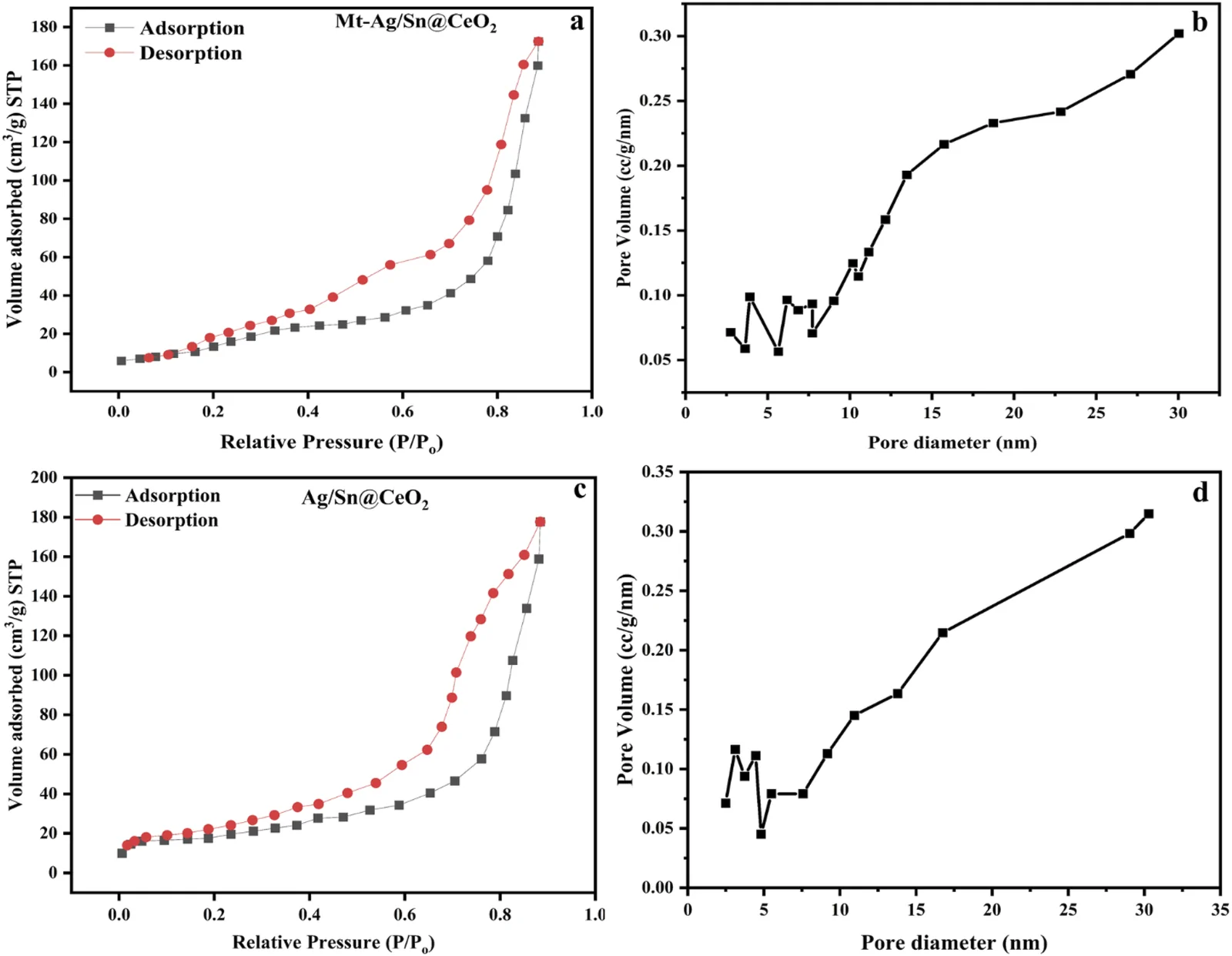
N_2_ adsorption–desorption isotherms and BJH pore size distribution of (a and b) Mt-Ag/Sn@CeO_2_ and (c and d) Ag/Sn@CeO_2_ composite.

### 3.7. Surface chemical states and composition

The chemical state and surface composition of Ag/Sn@CeO_2_ were analyzed through X-ray photoelectron spectroscopy (XPS), as shown in Fig. 7. The Ce 3d spectrum shown in Fig. 7a possesses the characteristic multiplet structure of CeO_2,_ where the components *v*, *v*′, *v*″, and *v*″ arise from the spin–orbit–split Ce 3d states and the relevant final-state multiplet features. The peak associated with Ce^4+^ shows high binding energy *v*″ and *u*″, while the peak associated with Ce^3+^ signifies that the amount of Ce^4+^ reduced at the surface is low. The spectrum of O 1s shown in Fig. 7b is represented by three components O_lattice_, O_defect_, and OH_ads,_ at 529.1 eV, 531.1 eV, and 532.5 eV, respectively. The considerable contribution made by defect-associated oxygen indicates that oxygen-deficient surface has formed. The Ag 3d spectrum shown in Fig. 7c has two peaks at Ag 3d_5/2_ at 368.3 eV and Ag 3d_3/2_ at 374.3 eV, indicating the presence of Ag. The Sn 3d spectrum shown in Fig. 7d indicates the presence of Sn 3d_5/2_ and Sn 3d_3/2_ peaks at around energies of 486.7 and 495.1 eV, respectively, with a peak separation of around 8.4 eV, which shows that predominantly Sn^4+^ species are present. The peak that appears at an energy of around 103 eV (see Fig. 7e) could be attributed to Si 2p coming from the silicate framework of montmorillonite, thus indicating that the clay-support structure remains intact after incorporation of the Ag/Sn-CeO_2_ phase.

**Fig. 7:**
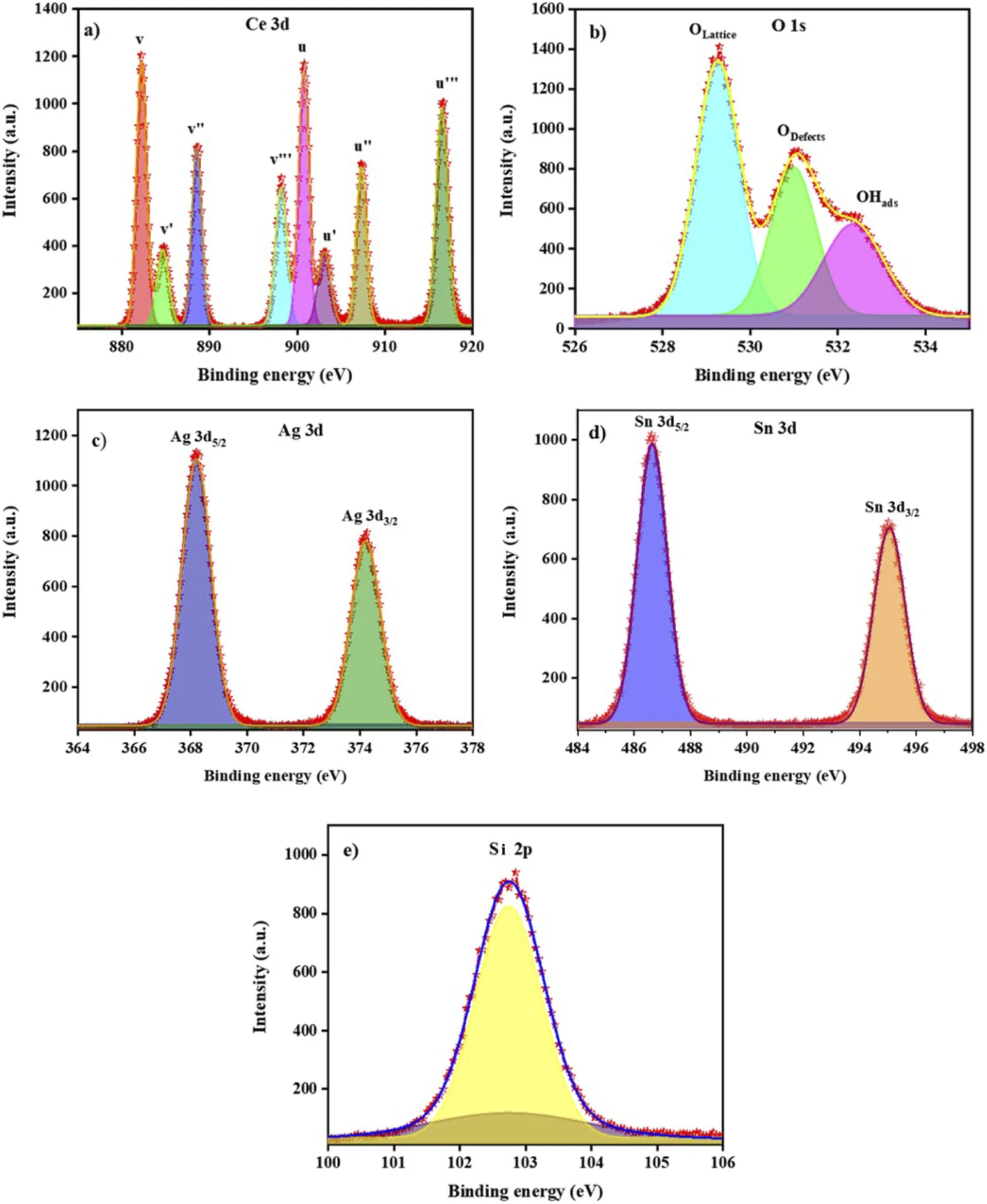
High-resolution XPS spectra of Mt-Ag/Sn@CeO_2_ showing the chemical states of (a) Ce 3d, (b) O 1s, (c) Ag 3d, (d) Sn 3d, and (e) Si 2p.

### 3.8. Dye adsorption analysis

Fig. 8 depicts the adsorption spectra of Ag/Sn@CeO_2_ and Mt-Ag/Sn@CeO_2_ composites, which reflect the considerable reduction in intensity of CV dye within a span of 180 minutes. The highly rapid initial adsorption indicates the abundance of active sites, and Mt-Ag/Sn@CeO_2_ showed better performance due to additional adsorption sites provided by montmorillonite.

**Fig. 8:**
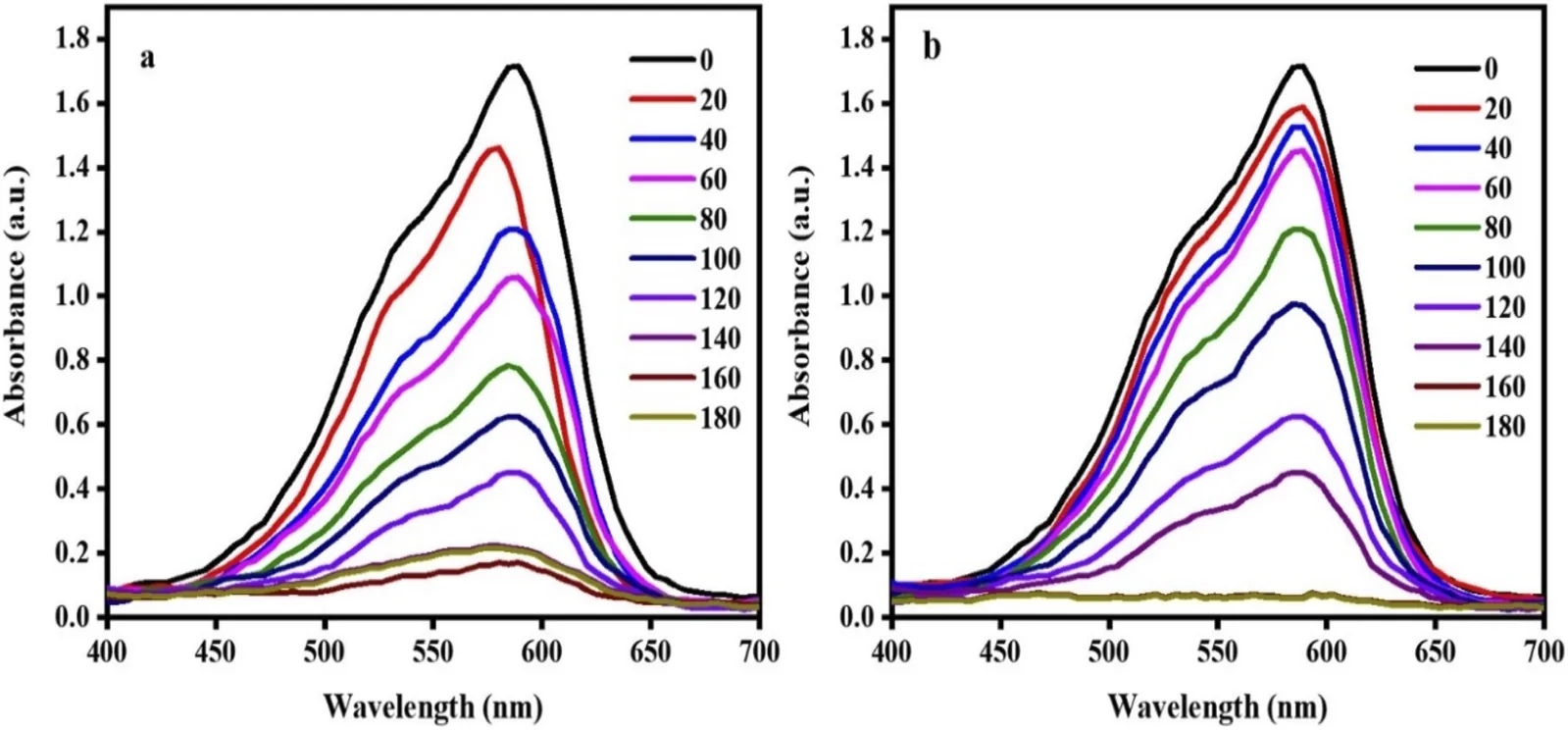
UV-visible spectra of the adsorption of CV on (a) Ag/Sn@CeO_2_ and (b) Mt-Ag/Sn@CeO_2_ composites.

### 3.9. Optimization of adsorption parameters

#### 3.9.1. Effect of pH

The solution pH has a significant impact on the adsorption behavior by altering the surface charge distribution of the adsorbent and modifying the ionization level of the dye molecules. The adsorption efficiency is affected by the nature and the strength of the interaction between the adsorbent surface and the dye ions.^45,49^ In this study, pH was varied from 4 to 10 while all other parameters were kept constant. The measured PZC of the composite is 6.5 (Section 3.5), which defines the switch point of surface charge. Below this value, protonation of surface hydroxyl groups imparts a net positive charge that repels cationic CV and suppresses uptake. Above it, deprotonation generates a net negative surface that favours electrostatic attraction with CV^+^, and the highest removal efficiency was accordingly recorded at pH 9–10. Although this attraction explains the pH-dependent trend in removal efficiency, the overall adsorption capacity remains moderate. This indicates that the electrostatic interaction is not the sole governing factor; non-electrostatic interactions, including π–π stacking, hydrogen bonding, and van der Waals forces, also contribute to the total uptake.^50,51^ As the pH increased from 4 to 10, the percentage removal rose from 38.30% to 89.22% for Ag/Sn@CeO_2_ and from 42.23% to 96.08% for Mt-Ag/Sn@CeO_2_ as shown in Fig. 9a.

**Fig. 9:**
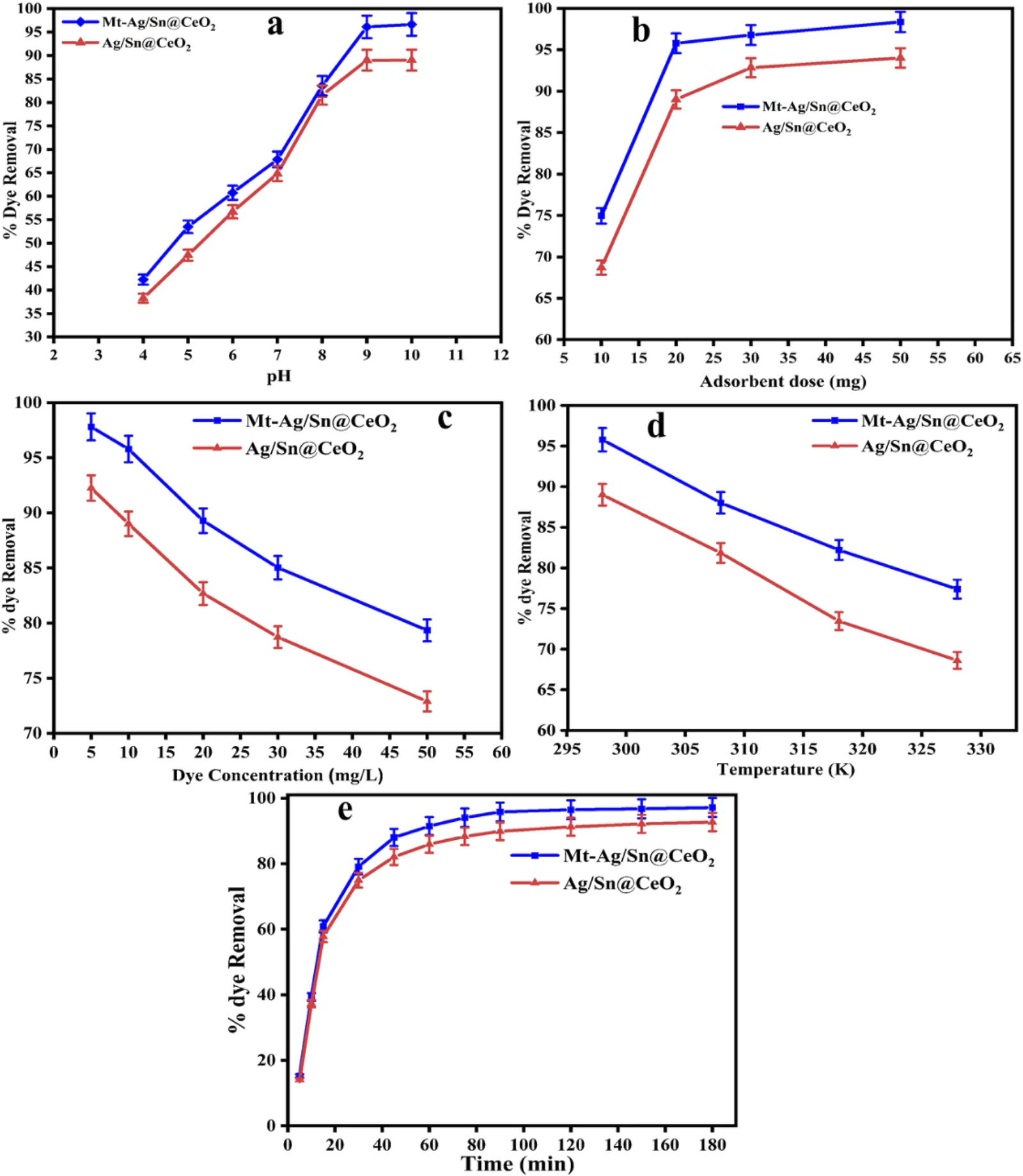
Effect of (a) pH, (b) adsorbent dosage, (c) dye concentration, (d) temperature, and (e) contact time on the removal percentage of CV dye. Unless varied, conditions were: adsorbent dose 20 mg, CV concentration 10 mg L^−1^, pH 9, temperature 298 K (error bars represent the SD of three independent measurements).

Overall, the synergistic effect of montmorillonite and Ag/Sn@CeO_2_ provides multiple active sites, with electrostatic attraction dominating at alkaline pH and contributing to the enhanced removal efficiency of CV.^52^

#### 3.9.2. Effect of adsorbent dosage

The effect of adsorbent dosage on the adsorption capacity of CV was observed using dosages ranging from 10 to 50 mg at an initial dye concentration of 10 mg L^−1^, as shown in Fig. 9b. The effect of adsorbent dosage on both composites was calculated based on their adsorption capacity and percentage removal. The percentage uptake of CV increased with rising adsorbent dosage, because the higher the adsorbent dose, the greater will be the available surface active sites.^53^ It was found that the percentage uptake of CV by Mt-Ag/Sn@CeO_2_ and Ag/Sn@CeO_2_ increased from 74.96% to 98.38% and from 68.70% to 94.02%, respectively. As the quantity increases, the adsorption process becomes more favorable, resulting in a reduction in equilibrium time. Beyond 20 mg, further increase in dosage did not significantly affect dye uptake, which remained nearly constant, indicating that the adsorption equilibrium had been reached. This is attributed to the excess of active sites compared to available CV molecules.^54^

#### 3.9.3. Effect of initial dye concentration

The initial concentration of dye provides the driving force for mass transfer between the liquid and solid phases. This enhances the transport of solute molecules across the liquid–solid interface, thus reducing the resistance to mass transfer.^52^ As the initial conc. of CV increased, a rise in adsorption capacity was accompanied by a decline in percentage removal, highlighting the dependency of CV adsorption on initial dye concentration (Fig. 9c). Increasing the initial CV dye amount from 5 mg L^−1^ to 50 mg L^−1^ corresponds to an increase in sorption capacities on Ag/Sn@CeO_2_ and Mt-Ag/Sn@CeO_2_ from 2.1 mg g^−1^ to 18.80 mg g^−1^ and 2.4 mg g^−1^ to 20.92 mg g^−1^, respectively.^55^ The opposite trends occur because a fixed mass of adsorbent has a limited number of active sites. At low concentrations, there are more vacant sites than dye molecules, so almost all the CV is removed. At higher concentrations, the sites become saturated, so a smaller fraction of the dye is adsorbed, although the absolute uptake per gram still increases because of the stronger concentration gradient.^56^ The maximum dye removal by Mt-Ag/Sn@CeO_2_ and Ag/Sn@CeO_2_ was found to be 97.8% and 92.30%, respectively, at optimum conditions.

#### 3.9.4. Effect of temperature

The impact of temperature (at 298, 308, 318, and 328 K) on the adsorption of CV was studied (Fig. 9d). The maximum adsorption efficiency was obtained at 298 K, reaching 95.6% for Mt-Ag/Sn@CeO_2_ and 89.2% for Ag/Sn@CeO_2_. With the temperature rising to 328 K, the adsorption efficiency declined to 77.8% and 68.51%, respectively. The decline in adsorption efficiency with increasing temperature further confirms the exothermic nature of the process, as indicated by the negative Δ*H*° values obtained from thermodynamic analysis. Higher temperatures reduce the strength of electrostatic and chemisorption interactions between CV molecules and the active sites of the adsorbent, while enhancing desorption. Although elevated temperature can lower aqueous viscosity and increase diffusion rates, these kinetic effects were insufficient to overcome the unfavorable thermodynamic shift, resulting in lower overall adsorption.^57,58^

#### 3.9.5. Effect of contact time

Reaction time plays a critical role in influencing the kinetics of the adsorption. In this study, its effect was evaluated at various intervals (0, 20, 40, 60, 80, 100, 120, 140, 160, and 180 minutes) while maintaining all other conditions constant. The adsorption of CV increased with prolonged contact time until equilibrium was reached, at which point all available adsorptive sites were saturated. As contact time increased, a higher removal percentage of CV was obtained. After 180 minutes, no further improvement in dye adsorption by either adsorbent was observed. The removal percentages reached 96.08% and 89.22% for Mt-Ag/Sn@CeO_2_ and Ag/Sn@CeO_2_, respectively, after 180 minutes of contact time, as shown in Fig. 9e.

### 3.10. Adsorption kinetics and thermodynamics

Kinetic investigations of crystal violet adsorption onto Ag/Sn@CeO_2_ and Mt-Ag/Sn@CeO_2_ composites were conducted to gain better insight into the adsorption mechanism (Fig. 10). The experimental kinetic data were interpreted using two commonly applied models: pseudo-first-order and pseudo-second-order kinetic models.

**Fig. 10:**
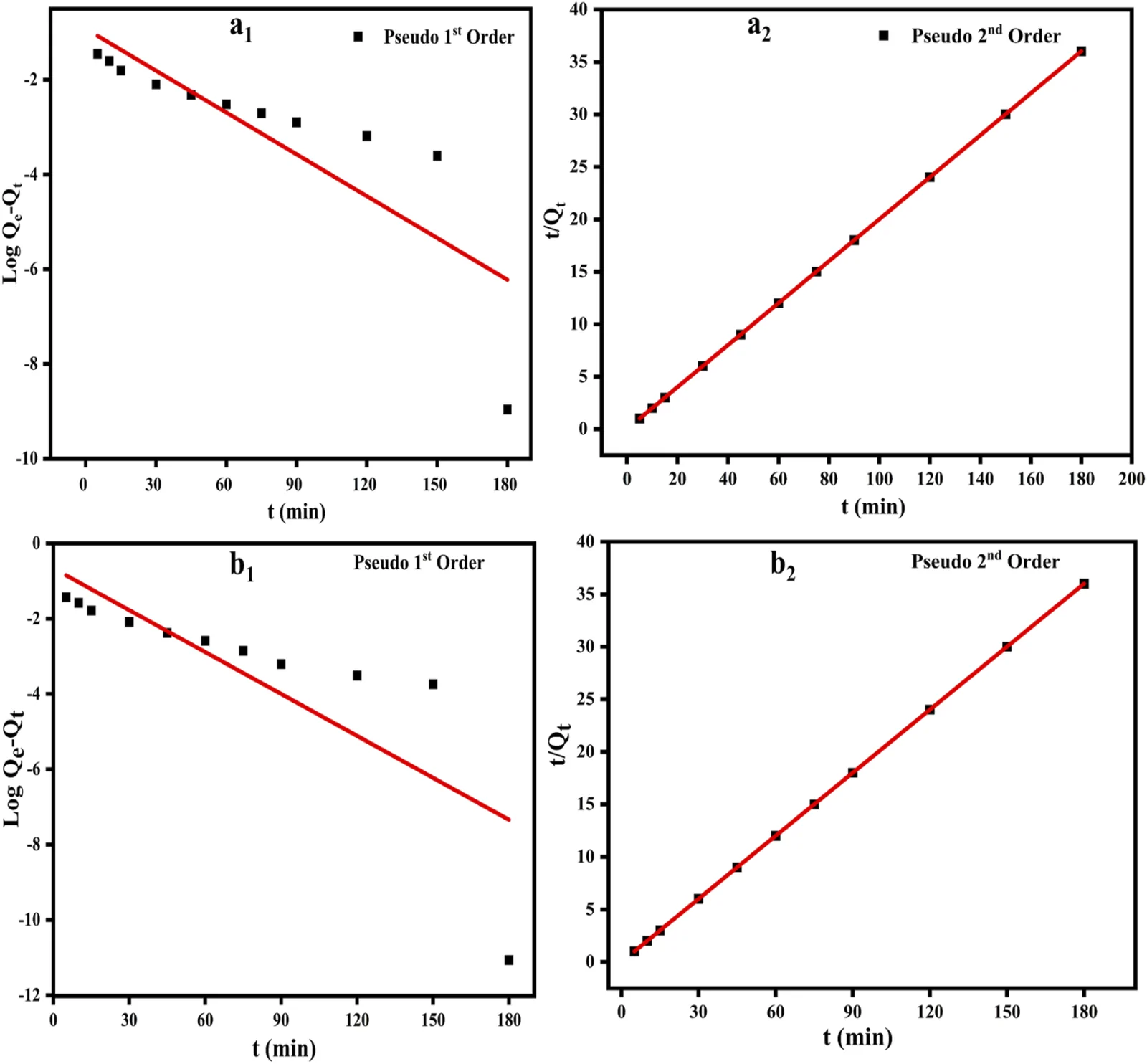
Kinetic model for the adsorption of dye using (a_1–2_) Ag/Sn@CeO_2_ and (b_1–2_) Mt-Ag/Sn@CeO_2_ composite.

The pseudo-first-order model postulates that the adsorption rate is directly proportional to the difference between the equilibrium adsorption capacity and the amount adsorbed at a given time. Its linearized and nonlinearized forms are provided by4
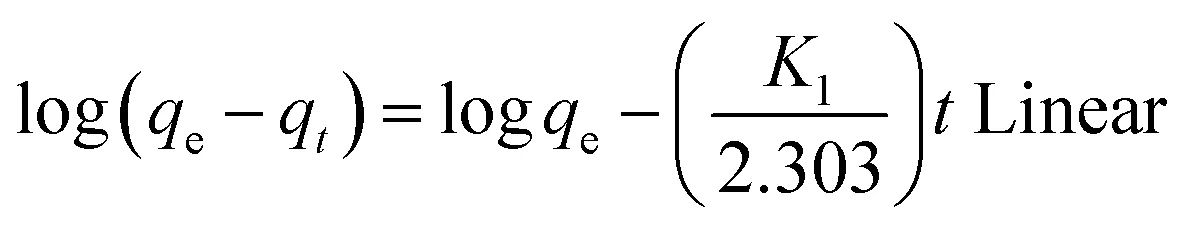

5 *q*_*t*_ = *q*_e_ (1 −*e*^−*K*_1_*t*^) Nonlinear

Here, *q*_*t*_ and *q*_e_ (mg g^−1^) denote the adsorption capacities at time *t* and at equilibrium, respectively; *K*_1_ (min^−1^) represents the rate constant for the pseudo-first-order model, and *t* is the contact time (min).^59^

The pseudo-second-order model assumes that adsorption mainly occurs through chemisorption, involving the sharing or exchange of electrons between dye molecules and the adsorbent surface.

Its linear and nonlinear forms can be written as eqn (6) and (7).6
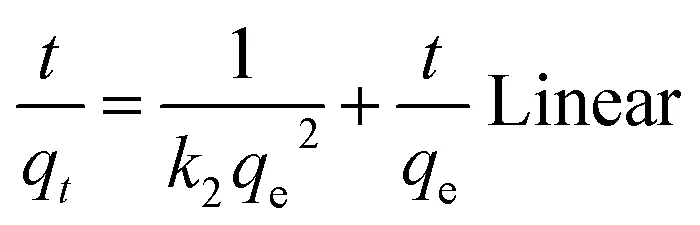
7
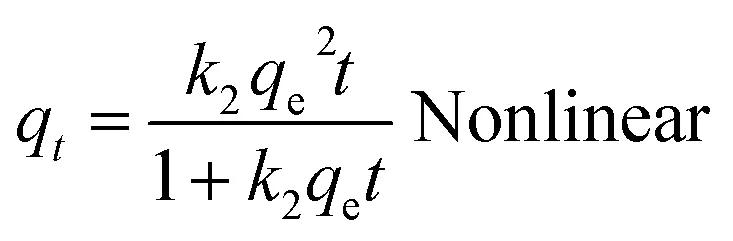
where *k*_2_ (g mg^−1^ min^−1^) is the pseudo-second-order rate constant.

The kinetic data for CV adsorption on Ag/Sn@CeO_2_ and Mt-Ag/Sn@CeO_2_ composites closely followed this model, showing a high *R*^2^ value and minimal difference between experimental and calculated *q*_e_ values (Table 1). The PSO model showed higher *R*^2^ values (0.958 and 0.960 for Mt and Ag/Sn, nonlinear) and lower error values compared to PFO. This agrees with a chemisorption-controlled process in which electrons are shared or exchanged between CV molecules and the surface sites. A better PSO fit alone is not sufficient proof of chemisorption, as this model can also fit systems controlled by other rate-limiting steps.^60^ The chemisorption interpretation is therefore supported here by the combined evidence of the kinetic fit, the magnitude of Δ*H*° (Section 3.10), and the surface interactions identified in the proposed mechanism (Section 3.11). To further evaluate the suitability of both kinetic models, error functions, including the sum of squared errors SSE, root mean square error RMSE and chi-square *χ*^2^ were computed in Table 1. Error functions were computed for the nonlinear fits only, as linearization distorts the error distribution and *R*^2^ is the conventional criterion for linear kinetic models.

**Table 1:** Adsorption kinetic models for the removal of CV dye

Model	Parameter	Mt-Ag/Sn@CeO_2_		Ag/Sn@CeO_2_	
		Linear	Non-linear	Linear	Non-linear
PFO	*Q*_e_ (mg g^−1^)	4.781	4.993	4.290	4.990
	*k*_1_ (1/min)	0.0376	1.012	0.0326	1.024
	*R* ^2^	0.930	0.566	0.947	0.569
	SSE	—	6.94 × 10^−4^	—	6.04 × 10^−4^
	RMSE	—	8.78 × 10^−3^	—	8.19 × 10^−3^
	*χ* ^2^	—	1.39 × 10^−4^	—	1.21 × 10^−4^
PSO	*Q*_e_ (mg g^−1^)	5.000	4.999	4.998	4.996
	*k*_2_ (g mg^−1^ min^−1^)	4.018	4.775	3.951	5.095
	*R* ^2^	0.945	0.958	0.953	0.960
	SSE	—	6.67 × 10^−5^	—	5.60 × 10^−5^
	RMSE	—	2.72 × 10^−3^	—	2.49 × 10^−3^
	*χ* ^2^	—	1.34 × 10^−5^	—	1.13 × 10^−5^

Temperature strongly influenced the adsorption of CV onto Ag/Sn@CeO_2_ and Mt-Ag/Sn@CeO_2_. As the temperature increased from 298 K to 328 K, both the adsorption capacity and equilibrium constant (*K*_L_) decreased, indicating that lower temperatures favour adsorption. The equilibrium constant *K*_L_ was obtained from the Langmuir isotherm fitting at each temperature. The values of Δ*H*° and Δ*S*° were determined from the slope and intercept of the plot of ln *K*_c_*versus* 1/*T*Fig. 11, and Δ*G*° was calculated at each temperature using the relationship Δ*G*° = Δ*H*° − *T*Δ*S*°. The enthalpy change (Δ*H*°) was negative for both the adsorbents (−69.90 kJ mol^−1^ for Mt-Ag/Sn@CeO_2_ and −53.91 kJ mol^−1^ for Ag/Sn@CeO_2_) in the non-linear plot. The negative value confirms the exothermic nature of CV adsorption. The magnitude of Δ*H*° also provides insight into the adsorption mechanism. Values below 40 kJ mol^−1^ are generally associated with physisorption, whereas values above this threshold indicate a significant contribution from chemisorption.^61^ The Δ*H*° suggests that chemisorption contributes considerably to CV uptake. This is further supported by the strong fit of experimental data to the pseudo-second-order kinetic model. The entropy change Δ*S*° was also negative (−208.7 J mol^−1^ K^−1^ for Mt-Ag/Sn@CeO_2_ and −116.64 J mol^−1^ K^−1^ for Ag/Sn@CeO_2_) in a non-linear plot. The negative value indicates a decrease in randomness at the solid–liquid interface during adsorption. It reflects the ordered arrangement of CV molecules on the adsorbent surface and the loss of their translational freedom upon adsorption.^62^ The Gibbs free energy change Δ*G*° was negative at all studied temperatures, confirming that the adsorption process is thermodynamically spontaneous. The Δ*G*° value became less negative as temperature increased, consistent with the exothermic nature of the adsorption process. The slightly more negative Δ*G*° and larger Δ*H*° magnitude for Mt-Ag/Sn@CeO_2_ compared to Ag/Sn@CeO_2_ indicate a stronger and more favourable interaction between CV and the Mt-based composite. Collectively, the negative values of Δ*H*°, Δ*G*°, and Δ*S*° confirm that CV adsorption onto both composites is spontaneous, exothermic, and enthalpy-driven (Table 2).

**Fig. 11:**
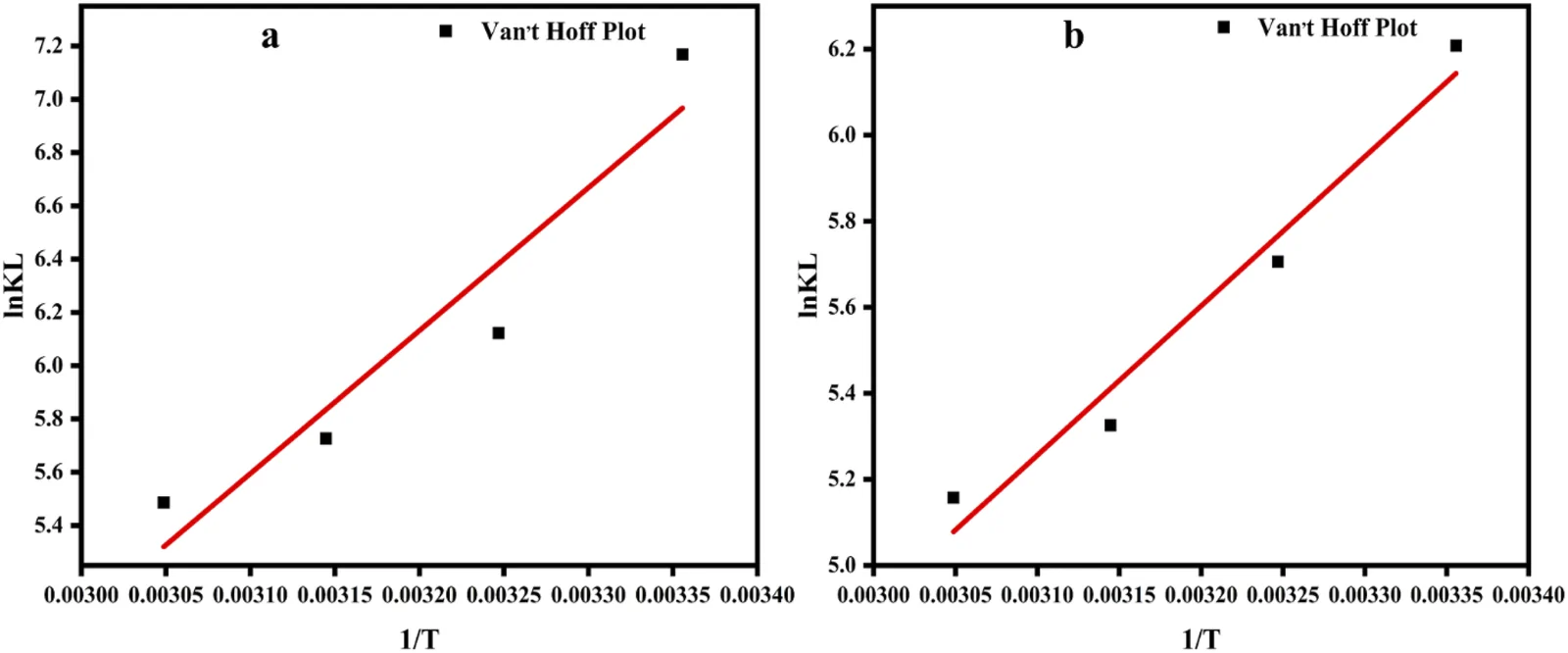
The plot of lnKL and 1/T for the adsorption of CV on (a) Ag/Sn@CeO_2_ and (b) Mt-Ag/Sn@CeO_2_ adsorbents.

**Table 2:** Thermodynamic parameters for CV adsorption^a^

Adsorbents	*T* (K)	*K*_L_	ln *K*_L_	Δ*G*°-L (kJ mol^−1^)	Δ*G*°-NL (kJ mol^−1^)	Δ*H*°-L (kJ mol^−1^)	Δ*H*°-NL (kJ mol^−1^)	Δ*S*°-L (J mol^−1^ K^−1^)	Δ*S*°-NL (J mol^−1^ K^−1^)	*R* ^2^-L	*R* ^2^-NL
Mt-Ag/Sn@CeO_2_	298	22.766	3.1253	−7.235	−7.703	−50.33	−69.90	−144.62	−208.71	0.977	0.974
	308	7.358	1.9958	−5.788	−5.615						
	318	4.627	1.5318	−4.342	−3.528						
	328	3.428	1.2319	−2.896	−1.441						
Ag/Sn@CeO_2_	298	8.106	2.0926	−5.029	−5.153	−43.02	−53.91	−104.00	−116.64	0.926	0.990
	308	4.514	1.5072	−3.989	−3.987						
	318	2.772	1.0197	−2.949	−2.820						
	328	2.190	0.7841	−1.909	−1.654						

a *K*_L_ is the Langmuir equilibrium constant obtained from isotherm fitting at each temperature. L = linear regression; NL = nonlinear regression.

#### 3.10.1. Adsorption isotherms

Adsorption studies were performed under controlled conditions, where the dye concentration ranged from 5 ppm to 50 ppm, the adsorbent dosage was fixed at 20 mg, and the pH was maintained at 9 with a temperature of 298 K (Fig. 12–14).

**Fig. 12:**
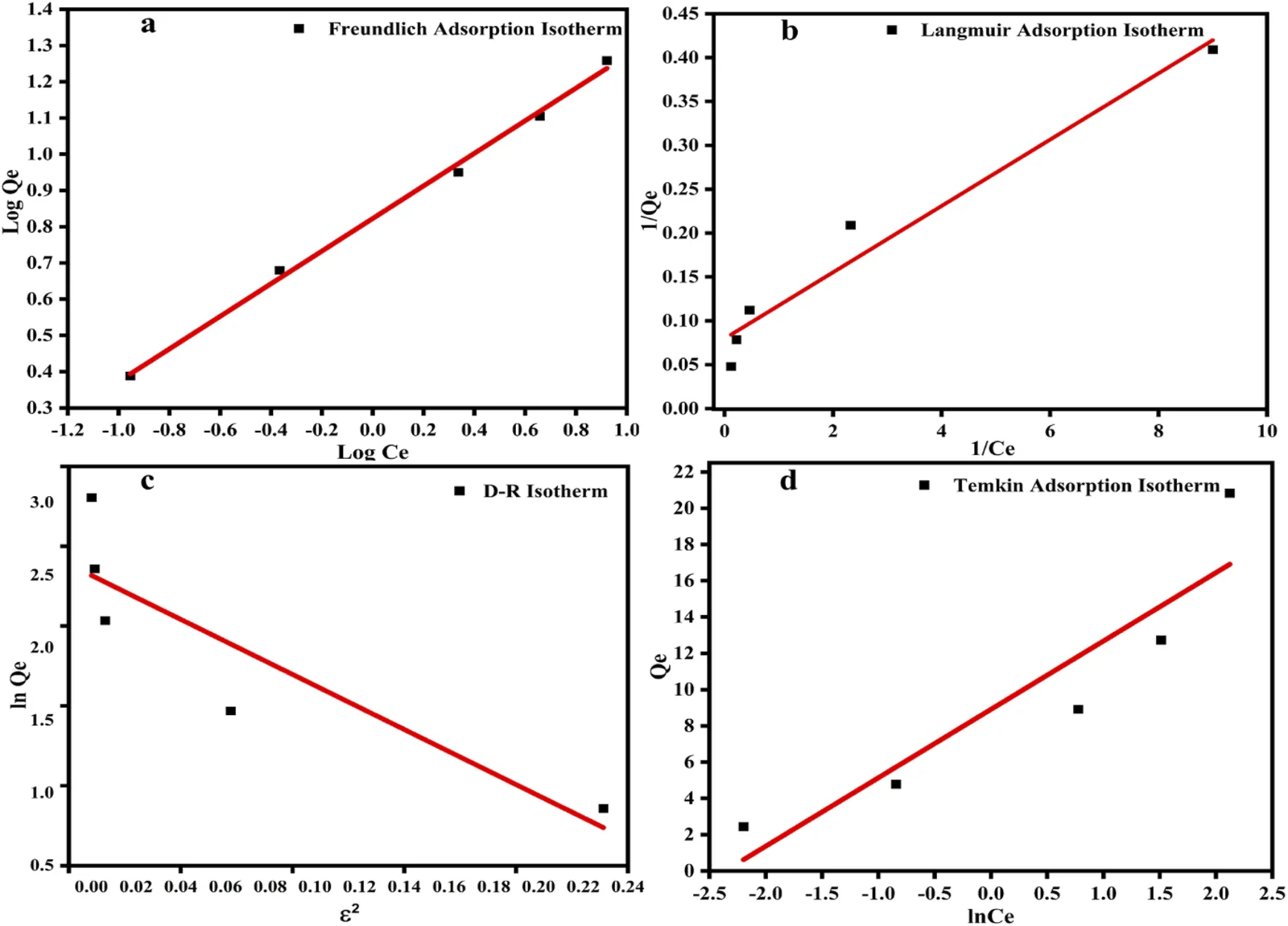
(a) Freundlich, (b) Langmuir, (c) D–R, and (d) Temkin adsorption linear isotherms of the adsorption of CV dye on Mt-Ag/Sn@CeO_2_ composite.

**Fig. 13:**
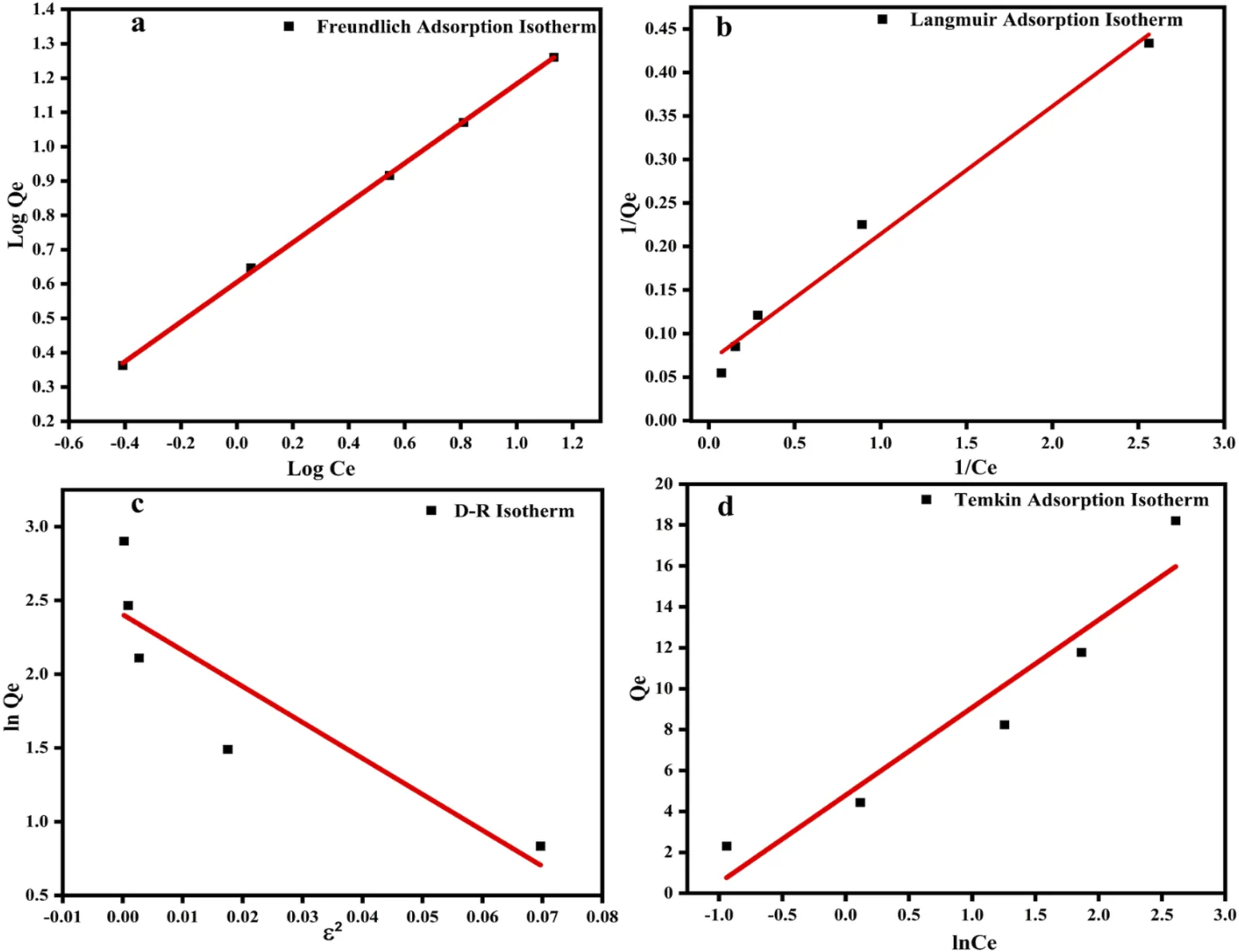
(a) Freundlich, (b) Langmuir, (c) D–R, and (d) Temkin adsorption linear isotherms of the adsorption of CV dye on Ag/Sn@CeO_2_ composite.

**Fig. 14:**
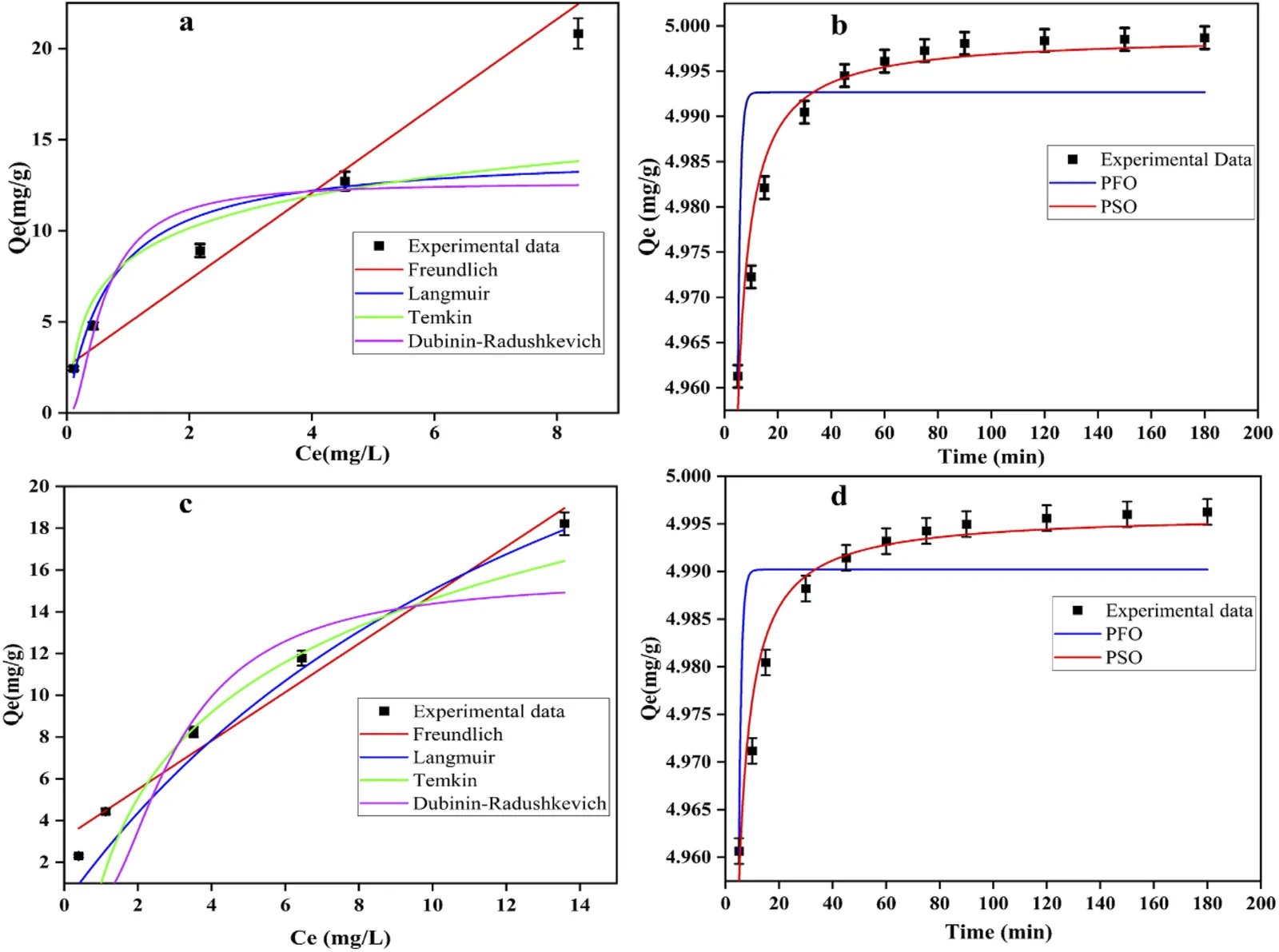
Nonlinear isotherms (Freundlich, Langmuir, Temkin, D–R) and kinetic (PFO, PSO) model fittings for dye adsorption onto (a and b) Mt-Ag/Sn@CeO_2_ and (c and d) Ag/Sn@CeO_2_ (error bars represent SD of three independent measurements).

The Langmuir isotherm model describes the relationship between the equilibrium concentration of adsorbate and the amount adsorbed, and it can be formulated in both nonlinear (8) and linear expressions (9).8
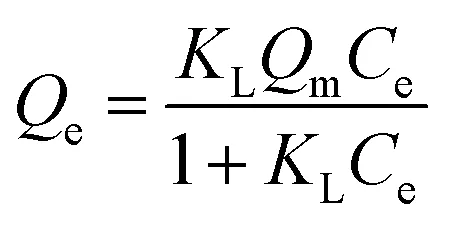
9
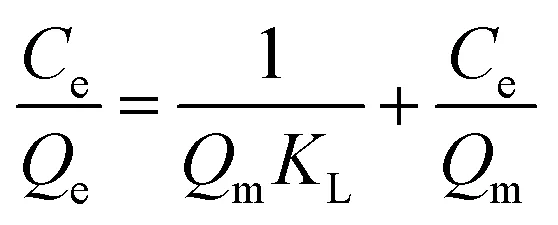


In the equation, *C*_e_ refers to the equilibrium concentration of adsorbate (mg L^−1^), *Q*_m_ refers to the maximum adsorption capacity (mg g^−1^), and *Q*_e_ represents the equilibrium adsorption capacity (mg g^−1^). The dimensionless separation factor (*R*_L_) evaluates the favorability of adsorption.10
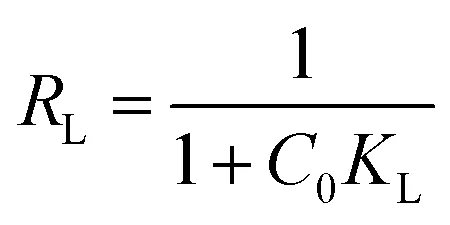


Values of 0 < *R*_L_ < 1 indicate favorable adsorption.where *R*_L_ is the dimensionless separation factor and *C*_0_ is the initial dye concentration (mg L^−1^). Values of *R*_L_ between 0 and 1 indicate favourable adsorption.

The Freundlich isotherm model explains adsorption on heterogeneous surfaces through a multilayer adsorption process.

11 ln *Q*_e_ = ln *K*_F_ + (1/*n*)ln *C*_e_

12 *Q*_e_ = *K*_F_*C*_e_^1/*n*^

where *K*_F_ (mg g^−1^) (L mg^−1^)^(1/*n*)^ is the Freundlich constant related to adsorption capacity, and *n* is the heterogeneity factor indicating adsorption favourability. A higher value of *R*^2^ indicates better model fitting.^63^

The Temkin model accounts for the effect of indirect adsorbate–adsorbent interactions and assumes that the adsorption heat decreases linearly with surface coverage. The linear and nonlinear form of the Temkin model was applied to fit the experimental data directly. The corresponding linear and nonlinear parameters are presented in Table 3.13
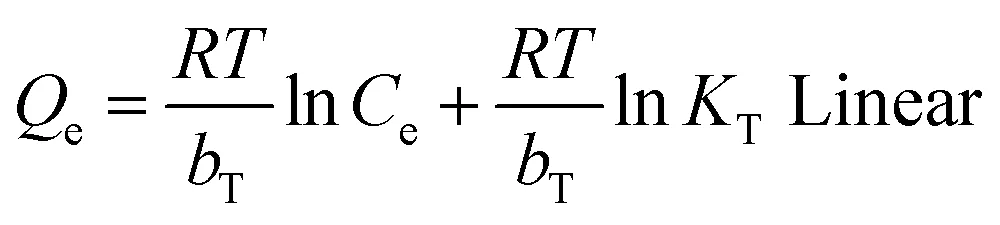

14 *Q*_e_ = *RT*/*b*_T_ ln(*A*_T_*C*_e_) Nonlinear

where *C*_e_ is the equilibrium concentration (mg L^−1^), *A*_T_ is the equilibrium binding constant (L mg^−1^), *b*_T_ is the Temkin constant related to the heat of adsorption (J mol^−1^), *q*_e_ is the equilibrium adsorption capacity (mg g^−1^), *R* is the universal gas constant (8.314 J mol^−1^ K^−1^), and *T* is the absolute temperature (K).^64^

**Table 3:** Different adsorption isotherm models for the removal of CV dye

Model	Parameter	Mt-Ag/Sn@CeO_2_		Ag/Sn@CeO_2_	
		Linear	Nonlinear	Linear	Nonlinear
Langmuir	*Q*_max_ (mg g^−1^)	23.36	36.50	23.94	28.77
	*K*_L_ (L mg^−1^)	0.457	0.145	0.191	0.120
	*R* ^2^	0.86	0.93	0.937	0.982
	SSE	24.623	14.28	5.26	2.91
	RMSE	2.865	2.182	1.324	0.985
	*χ* ^2^	3.278	9.522	0.660	1.174
Freundlich	*n*	2.127	1.771	1.731	1.729
	*K*_F_ (mg g^−1^)(L mg^−1^)^1/*n*^	6.79	6.009	4.031	4.023
	*R* ^2^	0.99	0.98	0.999	0.999
	SSE	7.84	4.53	0.029	0.028
	RMSE	1.617	1.229	0.098	0.097
	*χ* ^2^	0.492	0.780	0.006	0.006
Temkin	*A* (L mg^−1^)	10.61	10.61	3.058	3.058
	*B* (J mol^−1^)	3.77	3.77	4.286	4.286
	*R* ^2^	0.849	0.849	0.91	0.91
	SSE	31.63	31.63	12.86	12.86
	RMSE	3.247	3.247	2.071	2.071
	*χ* ^2^	7.378	7.378	4.026	4.026
Dubinin–Radushkevich	*E* (kJ mol^−1^)	17.061	19.616	16.02	18.68
	*β* (mol^2^ kJ^−2^)	0.0534	0.191	0.17	2.28
	*Q*_s_ (mg g^−1^)	12.188	18.28	11.07	15.62
	*R* ^2^	0.788	0.704	0.78	0.81
	SSE	—	42.94	—	29.85
	RMSE	—	3.783	—	3.154

The Dubinin–Radushkevich isotherm model characterizes biosorption on heterogeneous surfaces with Gaussian energy distributions and is suitable for intermediate concentrations. It describes the pore-filling mechanism and explains the multilayer physical adsorption driven by van der Waals forces. The D–R isotherm is a semi-empirical equation that helps in distinguishing between physical and chemical adsorption of metal ions.

The linear (eqn (15)) and nonlinear (eqn (16)) forms of the D–R isotherm are

15 ln *q*_e_ = ln *q*_DR_ − *βε*^2^

16 *Q*_e_ = *Q*_m_ exp(−*βε*^2^)

where *ε* is the Polanyi potential defined by (eqn (17)) and the mean sorption-free energy *E*_s_ indicates whether the process is physical or chemical adsorption (eqn (18)).17
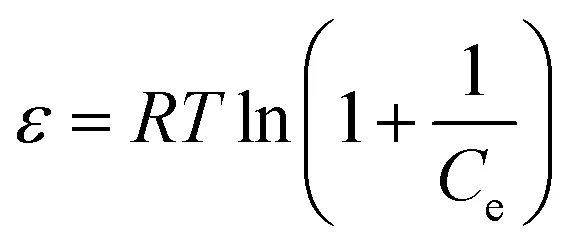
18
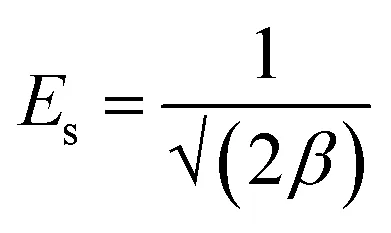
where *q*_DR_ is the theoretical saturation capacity (mg g^−1^), *β* is the activity coefficient related to adsorption energy (mol^2^ kJ^−2^), *ε* is the Polanyi potential, and *E* is the mean free energy of adsorption (kJ mol^−1^).

In this study, all four isotherm models were fitted using linear and nonlinear regression forms. The Freundlich model showed the best fit in both linear (*R*^2^ = 0.99 for Mt-Ag/Sn@CeO_2_ and 0.999 for Ag/Sn@CeO_2_) and nonlinear (*R*^2^ = 0.978 and 0.9998, respectively) regression forms, suggesting heterogeneous surface adsorption and multilayer dye uptake. The fitted data for these isotherm models are shown in Table 3.

To further evaluate the suitability of each isotherm model, error functions including the sum of squared errors SSE, root mean square error RMSE and chi-square *χ*^2^ were computed for both linear and nonlinear regression forms Table 3.

For both adsorbents, the Freundlich model yielded the lowest error values, confirming it as the best-fit isotherm. For Mt-Ag/Sn@CeO_2_, the Freundlich model gave SSE = 4.53, RMSE = 1.229, *χ*^2^ = 0.780, while for Ag/Sn@CeO_2_ the corresponding values were markedly lower (SSE = 0.028, RMSE = 0.097, *χ*^2^ = 0.006). These results are consistent with the high *R*^2^ values, confirming multilayer adsorption on a heterogeneous surface. Furthermore, the nonlinear regression form consistently produced lower error values than its linearized counterparts. This demonstrates that nonlinear regression provides more reliable parameter estimation, as the linearization of the isotherm equation distorts the error distribution.^65,66^ Although the Freundlich model fits the data best, the maximum adsorption capacity reported in this work is taken from the Langmuir model. The Freundlich equation has no saturation term, so it cannot give a single capacity value for comparison. Langmuir *Q*_max_ provides this and allows direct comparison with other adsorbents Table 5. The two models thus serve different purposes here: Freundlich describes how CV binds to the heterogeneous surface, while *Q*_max_ is used only as a benchmark and does not imply monolayer coverage.

### 3.11. CO_2_ adsorption study

Mt-Ag/Sn@CeO_2_ was selected for the adsorption of CO_2_ from a gas stream. The selection was based on its high adsorptive potential for CV dye. The CO_2_ adsorption ability of Mt-Ag/Sn@CeO_2_ was thoroughly investigated, and the results suggest that Mt-Ag/Sn@CeO_2_ has a large affinity for CO_2_, especially at high pressure. The definite quadrupole moment and polarizability of CO_2_ were demonstrated by the behavior of Mt-Ag/Sn@CeO_2_ to increase interactions with the exchangeable cations of the clay framework. Additionally, the improved gas trapping at high pressure led to improved CO_2_ retention, made possible by the sustained pore volume and three-dimensional porous network of the composite. The increase in pressure also enhanced these interactions, which consequently improved total adsorption capacity.^67^ The CO_2_ molecules interact with the layer cations and/or interlayer water molecules of the montmorillonite structure to start the total sorption process. The surface area, pore size, and pore volume of Mt-Ag/Sn@CeO_2_ influenced the CO_2_ adsorption capacity.^68^ The binding affinity for CO_2_ was also influenced by the presence of oxygen-centered functionalities, such as hydroxyl and carboxyl groups, which enhanced CO_2_ sorption due to the increased electron density around the oxygen atoms of the modified surface. Thus, the CO_2_ adsorption mechanism of Mt-Ag/Sn@CeO_2_ involves interactions with water molecules present in the interlayers of montmorillonite.^69,70^

As illustrated in Fig. 15, the adsorption pattern increases monotonically with pressure at both 298 and 313 K. This is consistent with general gas adsorption principles, which suggest that increased pressure pushes more gas molecules into the available pore spaces. The data indicated higher *k* values (0.0039 mmol g^−1^ mmHg^−1^) at 298 K compared to 313 K (0.0033 mmol g^−1^ mmHg^−1^), demonstrating greater adsorption at lower temperature. The *R*^2^ values are 0.9664 at 298 K and 0.9769 at 313 K. CO_2_ adsorption is exothermic, leading to desorption at higher temperatures. The comparison of CO_2_ adsorption by the Mt-Ag/Sn@CeO_2_ composite with other reported clay-based adsorbents is given in Table 4.

**Fig. 15:**
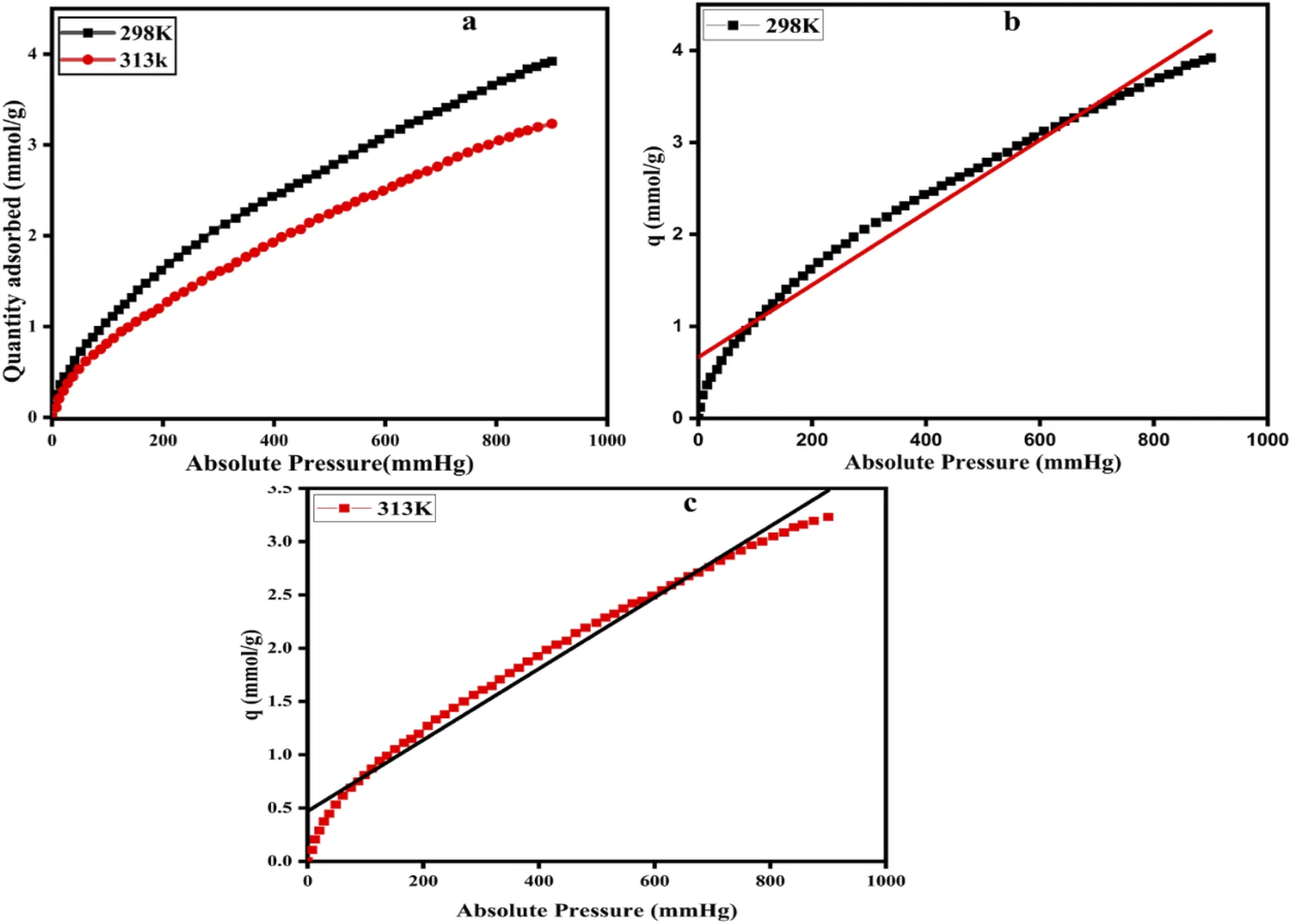
(a) CO_2_ adsorption isotherms and linear fits for Mt-Ag/Sn@CeO_2_ composite at (b) 298 K and (c) 313 K.

**Table 4:** Comparison of CO_2_ adsorption of Mt-Ag/Sn@CeO_2_ composite with other reported clay-based adsorbents

Material	Temperature (^°^C)	Adsorption capacity (mmol g^−1^)	Pressure	Reference
Amine-clay	25	1.46	1 atm	71
Raw sepiolite	25	1.48	760 mmHg	72
Sep-20 A40 P	65	2.07	760 mmHg	72
Sodium-montmorillonite	25	2.38	9 bar	73
PMMT/rGO	25	0.49	900 mmHg	67
Modified montmorillonite with triethanolamine	40	5.64	7 bar	74
Mt-Ag/Sn@CeO_2_	25	3.92	900 mmHg	Present study

### 3.12. Adsorptive mechanism

The adsorption behaviour of CV onto Mt-Ag/Sn@CeO_2_ composite occurs through several cooperative mechanisms (Fig. 16). First, electrostatic attraction plays a dominant role because CV is a cationic triphenylmethane dye (CV^+^). At the same time, Mt possesses a permanent negative surface charge arising from isomorphic substitution within its layered structure. At pH levels higher than the PZC, the negative surface charge density of the composite increases, thereby enhancing the electrostatic interaction between the negatively charged surface and positively charged CV^+^ ions.^42–44^ Secondly, the aromatic rings of CV are capable of π–π interactions and form a network with the π-electron-rich siloxane surfaces of montmorillonite and with Sn/CeO_2_ nanoparticles, thereby stabilizing the adsorbed dye molecules and enabling multilayer adsorption at higher dye concentrations. In addition, surface hydroxyl groups (≡Si–OH, ≡Al–OH, and –OH groups) contribute to hydrogen bonding with the nitrogen atoms of CV and its aromatic C–H groups, providing secondary stabilization, especially under near-neutral conditions.^75,76^ For the oxide component, the metal–oxide surfaces carry protonated –MOH_2_^+^ sites below their PZC, enhancing attraction toward anions but becoming neutral/negative above the PZC; reported PZC values place CeO_2_ roughly in the ∼6–8 range, aligning with the general PZC trends.^77^ In the Mt-supported mixed-oxide adsorbent, the clay acts as a high-area, negatively charged scaffold that disperses the nanoparticles, limits aggregation, and exposes active sites, a support effect widely reported for Mt-immobilized oxides (*e.g.*, Mt-Ag/SnO_2_) and for Mt-based pollutant removal.^78^ The comparison of different clay-based adsorbents for the adsorption of cationic dyes is given in Table 5. Taken together, these literature-backed interactions (electrostatic attraction of CV to Mt, π–π/H-bond contributions, and improved dispersion/active-site exposure on Mt supports) are consistent with the observation that the Mt-Ag/Sn@CeO_2_ composite outperforms Ag/Sn@CeO_2_ alone. The pH data give the clearest evidence for electrostatic attraction, since removal tracks the change in surface charge across the PZC of 6.5. For hydrogen bonding, the FTIR spectrum shows a broad O–H band at 3460–3410 cm^−1^, confirming that ≡Si–OH and ≡Al–OH groups are present and can interact with the nitrogen atoms of CV.^75,76^ Furthermore, the extended aromatic/conjugated structure of CV may allow π–π interactions with suitable π-electron-rich domains of the adsorbent. However, the Freundlich fit and the *n* value of 1.77 point to multilayer uptake, which agrees with dye molecules stacking above the first adsorbed layer. The Δ*H*° value of −69.90 kJ mol^−1^ is also too high for van der Waals forces alone and suggests that specific chemical interactions are involved.^61^ These interactions may operate simultaneously and contribute to the overall adsorption process. However, because post-adsorption spectroscopic measurements were not performed, the hydrogen-bonding and π–π interactions are regarded as plausible contributing interactions rather than individually verified mechanisms.

**Fig. 16:**
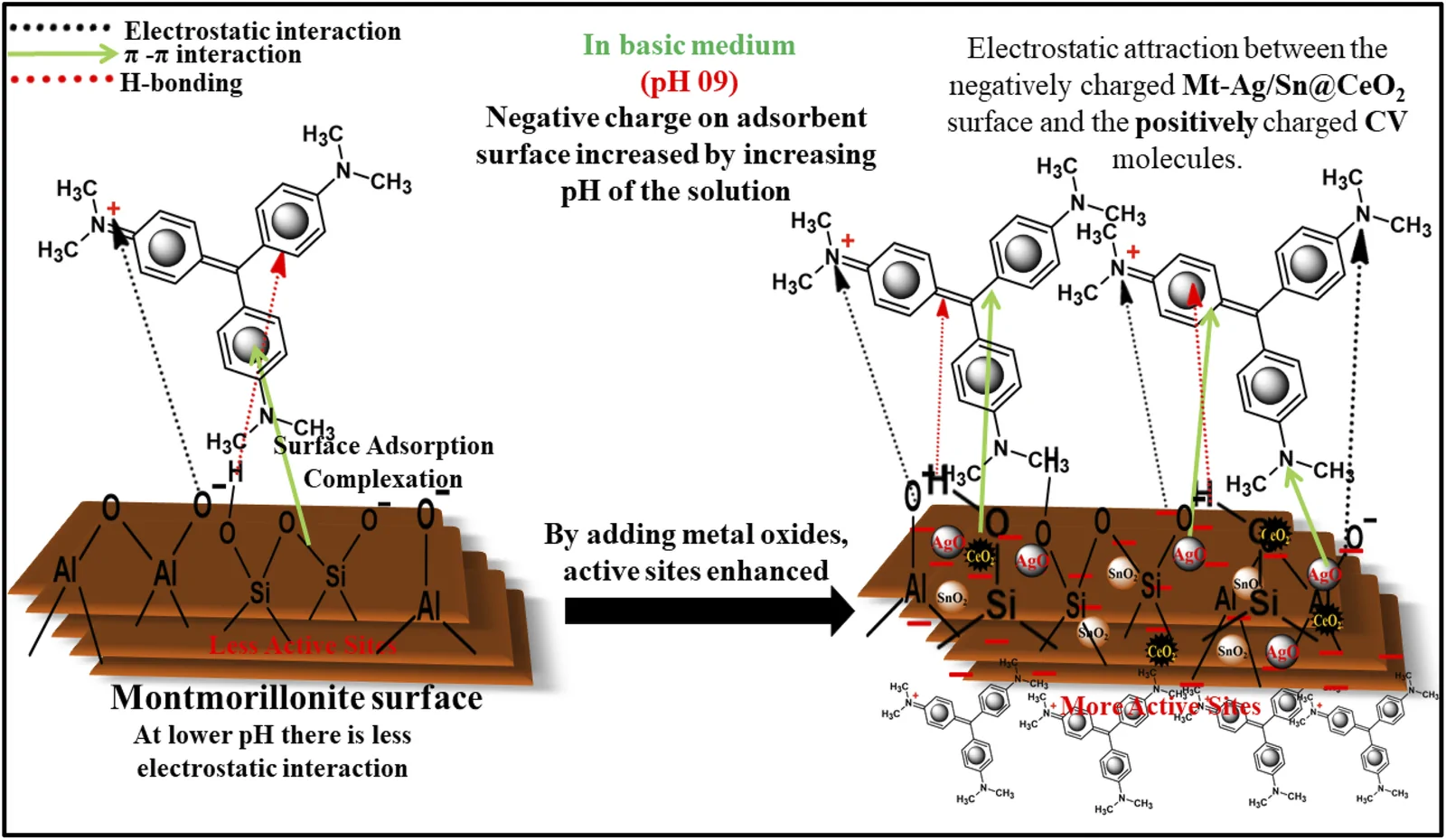
Schematic representation of CV dye adsorption on Mt-Ag/Sn@CeO_2_ composite.

**Table 5:** Comparison of different clay-based adsorbents for the adsorption of cationic dyes

Adsorbent	Adsorbent dosage	Maximum capacity mg g^−1^	Dye	Reference
(GO-MMT)	—	746.27 mg g^−1^	Crystal violet	79
Kaolin-organic bentonite	2.0 g	12.68 mg g^−1^	Rhodamine B	79
γ-Fe_2_O_3_@Mt	1 g L^−1^	209.20 mg g^−1^	Rhodamine B	80
CMC-g-(AA-co-IA)/MMT hydrogel composite	2 g L^−1^	19.12 mg g^−1^	Safranin-O	81
Bentonite/SDBS		25.97 mg g^−1^	Methylene blue	82
Activated bentonite–alginate composite	—	780 mg g^−1^	Methylene blue	83
Chemically modified MT nanoclay	0.4 g L^−1^	73.2 mg g^−1^	Basic green 4	39
Clay/starch/MnFe_2_O_4_ magnetic composite	1 g L^−1^	79.81 mg g^−1^	Sunset yellow (SY)	84
Ag/Sn@CeO_2_	2 g L^−1^	28.77 mg g^−1^	Crystal violet	Present study
Mt-Ag/Sn@CeO_2_	2 g L^−1^	36.50 mg g^−1^	Crystal violet	Present study

### 3.13. Regeneration and reusability

The reusability of both adsorbents was evaluated over five consecutive adsorption–desorption cycles, and the results are presented in Fig. 17a. For Mt-Ag/Sn@CeO_2_, the removal efficiency decreased only from 96.08% in the 1st cycle to 88.7% in the 5th cycle. In comparison, Ag/Sn@CeO_2_ showed a slightly larger decline from 89.22% to 79.5%. The Mt containing composite therefore retained 92.3% of its initial efficiency after five cycles, compared with 89.1% for the bare oxide. Desorption of CV was carried out using 0.1 M HCl, which has been reported as the most effective desorbing agent among common acids, bases, and salt solutions.^85^ Under acidic conditions, the surface becomes protonated and positively charged. This weakens the attraction to cationic CV and releases it from the surface. This behaviour agrees with the pH-dependent mechanism discussed earlier. The slight decline across cycles is common due to incomplete desorption and minor adsorbent loss during washing.^86^

**Fig. 17:**
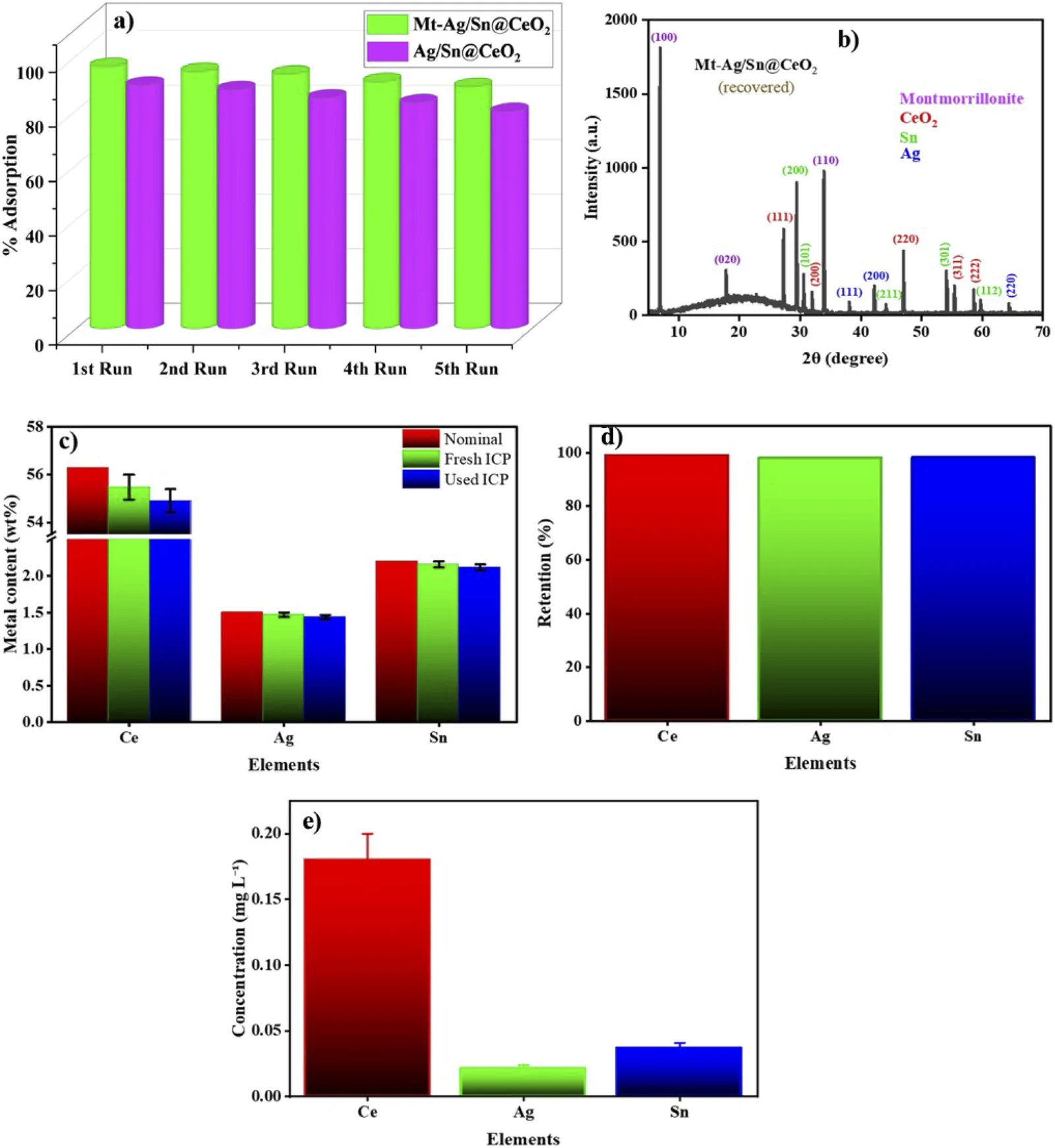
(a) Reusability of Mt-Ag/Sn@CeO_2_ and Ag/Sn@CeO_2_ composites over five adsorption–desorption cycles for CV removal, (b) post-adsorption XRD and (c–e) ICP-OES analysis of Mt-Ag/Sn@CeO_2_ composite.

According to post-adsorption XRD pattern (Fig. 17b), it is evident that there are no deformations in the structure of the adsorbent Mt-Ag/Sn@CeO_2_ after the CV removal. The characteristic peaks associated with the ceria fluorite crystals like (111), (200), (220), (311), (222), and (400) remain visible in the characterization results along with the peaks of montmorillonite. There are no apparent new peaks attributed to a new modified phase caused by the dye adsorption. Thus, regarding the used material, it is noticeable that individual reflections show some variations after the dye impact, the intense background significantly changes, especially in small angles, which can be explained by the presence of the dye molecules. More importantly, the main reflections of the XRD analysis show that the adsorption does not have any serious influence on the structural features of Mt-Ag/Sn@CeO_2_.

The elemental composition and stability of Mt-Ag/Sn@CeO_2_ were also studied through inductively coupled plasma-optical emission spectrometry (ICP-OES) (Fig. 17c–e). The fresh catalyst composition showed 55.5, 1.47, and 2.15 wt% concentrations of Ce, Ag, and Sn respectively, which represent nearly the same nominal values of 56.3, 1.50, and 2.20 wt%. The total recovery of the elemental content of the synthesized catalyst is nearly about 98%. Following experimentation, the recovered catalyst contained 54.9 wt% Ce, 1.44 wt% Ag, and 2.13 wt% Sn, which corresponds to the 97–99% of element content recovered compared to its fresh form. This small variance in the bulk elemental composition indicates the compositional stability of the co-catalyst used in the reaction. At last, the ICP-OES results from the post-reaction solution indicated that there were insignificant amounts of released Ce compounds (0.18 mg L^−1^), Ag compounds (0.021 mg L^−1^), and Sn compounds (0.038 mg L^−1^), which meant that only a minimal amount of the active components was able to dissolve or migrate to the reaction mixture. In this regard, the significant correlation between the nominal and observed values of the compounds along with the high stability of the elements after catalysis and the insignificant amount of leaching supports the conclusion that there is a very good immobilization and compositional stability of the CeO_2_ phase doped with Ag and Sn on montmorillonite.

## 4. Conclusion

In this work, we have demonstrated the successful synthesis of a silver/tin co-doped CeO_2_ nanostructure integrated with montmorillonite *via* a continuous-flow mechanochemical route. The nanoscale incorporation of Ag and Sn within the ceria lattice, combined with the layered architecture of montmorillonite, led to synergistic increases in surface area, active adsorption sites, and interfacial reactivity. These structural features directly contributed to the efficient removal of crystal violet dye and the effective capture of CO_2_ under ambient conditions. Characterization results confirmed the synthesis and indicated a porous structure with optimal incorporation of metallic cations within Mt layers. The analyses revealed that both composites were crystalline in nature, with the average crystallite size increasing from 40.76 to 53.16 nm due to the incorporation of montmorillonite to form (Mt-Ag/Sn@CeO_2_). To elucidate the precise mechanism of dye adsorption, various kinetic models and adsorption isotherms were employed. The pseudo-second-order kinetic model, along with the Freundlich model, provided the best correlation with the experimental data. The incorporation of montmorillonite enhanced surface functionality and active site availability, improving CV adsorption under optimized conditions. The Mt-Ag/Sn@CeO_2_ showed a maximum CV adsorption capacity of 36.50 mg g^−1^ compared to 28.77 mg g^−1^ for Ag/Sn@CeO_2_, due to the higher availability of surface functional groups for electrostatic interaction with the dye. Mt-Ag/Sn@CeO_2_ demonstrates a significant adsorption capacity of 3.92 mmol g^−1^ for CO_2_ from a gas stream.

Overall, the green mechanochemical fabrication of Mt-Ag/Sn@CeO_2_ provides a low-cost and scalable route to a dual-function adsorbent for water and air remediation.

### 4.1. Future perspectives

The present study establishes the adsorption potential of Mt-Ag/Sn@CeO_2_ under controlled laboratory conditions. Fixed-bed column studies are necessary to determine breakthrough curves, bed capacity at the pilot scale. Additionally, the CO_2_ adsorption selectivity of Mt-Ag/Sn@CeO_2_ in the presence of competing gases such as N_2_, CH_4_, and SO_2_ should be quantified using mixed-gas isotherm measurements. The effect of humidity on CO_2_ uptake capacity also requires systematic investigation, as moisture is inevitably present in industrial flue gas streams. Surface modification strategies such as amine functionalization could further enhance CO_2_ affinity and should be explored in follow-up studies. Finally, a life cycle assessment of the mechanochemical synthesis route is recommended to quantify its environmental and economic advantages over the conventional method.

## Author contributions

M. R., M. S., M. B. T.: conceptualization, methodology, writing – original draft preparation; A. M. A., K. A. T., M. M. A., A. A. A., R. A.: formal analysis; M. R., I. B.: data curation, software; M. B. T.: supervision, writing – reviewing and editing, M. B. T., F. V., R. A.: project administration, writing – reviewing and editing. All authors reviewed the manuscript.

## Conflicts of interest

The authors declare that they have no known competing financial interests or personal relationships that could have influenced the work reported in this paper.

## Supplementary Material

RA-OLF-D6RA07930J-s001

## Data Availability

The datasets generated and/or analyzed during the current study are openly available in the Open Science Framework (OSF) repository at https://osf.io/dq6ue/overview. This includes all raw data, processed data, and analysis scripts supporting the findings of this study. Supplementary information (SI): *Melothria scabra* leaf extract preparation, calibration curve for CV dye solution, and maximum absorbance (*λ*_max_) of CV dye. See DOI: https://doi.org/10.1039/d6ra07930j.
