# Supplementary material for: Montmorillonite-supported Ag/Sn@CeO2via phyto-mechanochemical engineering: enhanced crystal violet adsorption and CO2 capture
Source: RSC Adv. 2026 Oct 19;16(48):64498–518. doi: 10.1039/d6ra07930j (PMC13679713; doi:10.1039/d6ra07930j)
Supplement: Supplementary file 1 [file RA-016-D6RA07930J-s001.pdf]

Supplementary Information (SI) for RSC Advances.
This journal is © The Royal Society of Chemistry

Supplementary Materials

# Montmorillonite-supported Ag/Sn@CeO₂ via phyto-mechanochemical engineering: enhanced crystal violet adsorption and CO₂ capture

## 1.1 *Melothria Scabra* leaf extract

To prepare the extract from *Melothria scabra* leaves, fresh leaves were sourced from a local farm. They were thoroughly washed with tap water, followed by distilled water to eliminate dust and surface contaminants. After air-drying in the shade at room temperature for 3 days, the leaves were ground into a fine paste using a pestle and mortar. In a beaker, 100 mL of distilled water was mixed with 30 g of leaf paste, and the mixture was magnetically stirred at 70 °C for 1 hour. A color change was observed from green to brownish-yellow. The extract was then allowed to cool down, filtered using Whatman filter paper 01, and stored at 4°C for further use. The UV-visible spectrum of *Melothria scabra* leaf extract is shown in Fig. S1.

Figures

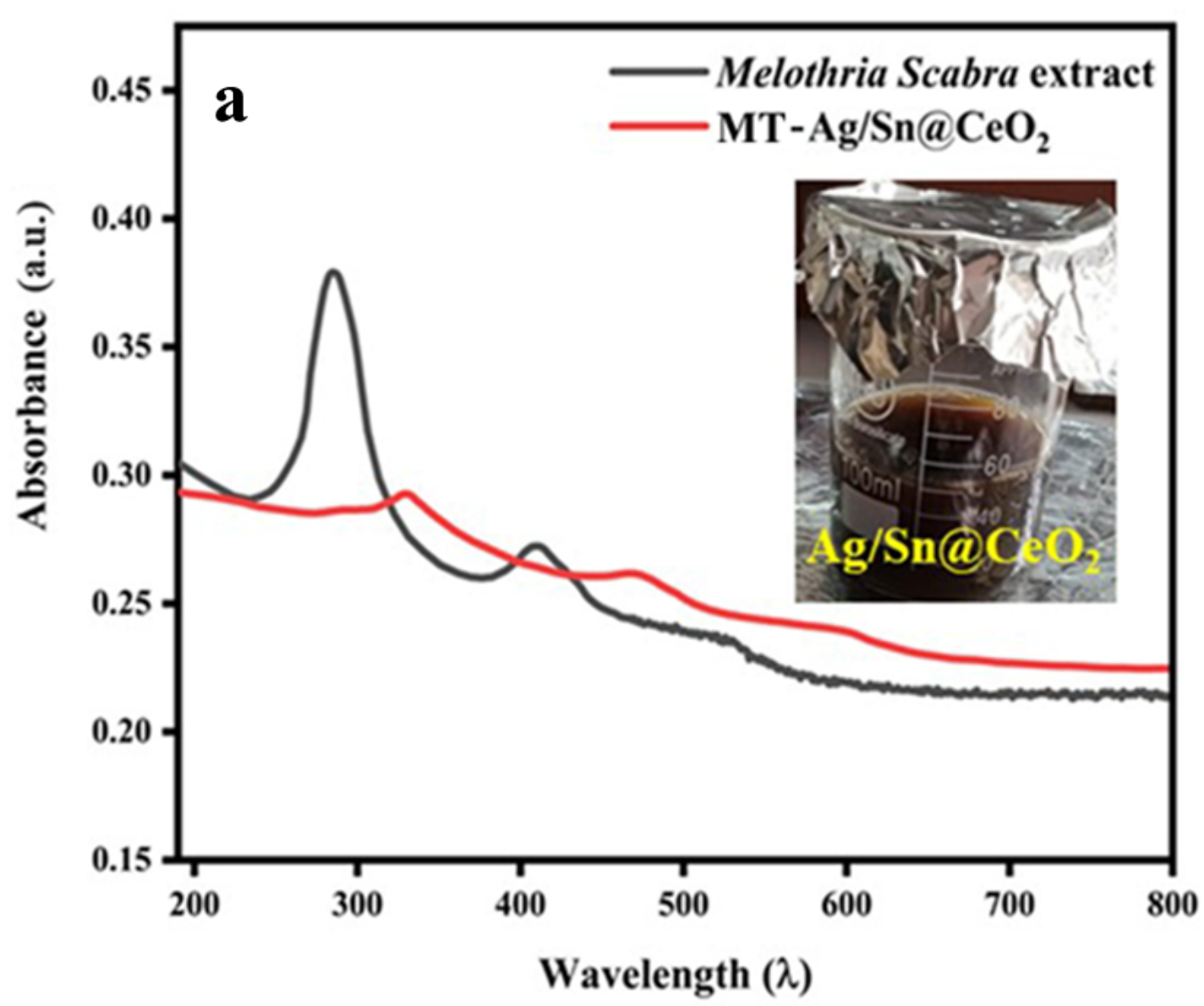


**Figure S1.** UV-visible spectra of *Melothria Scabra extract* and MT-Ag/Sn@$CeO_2$

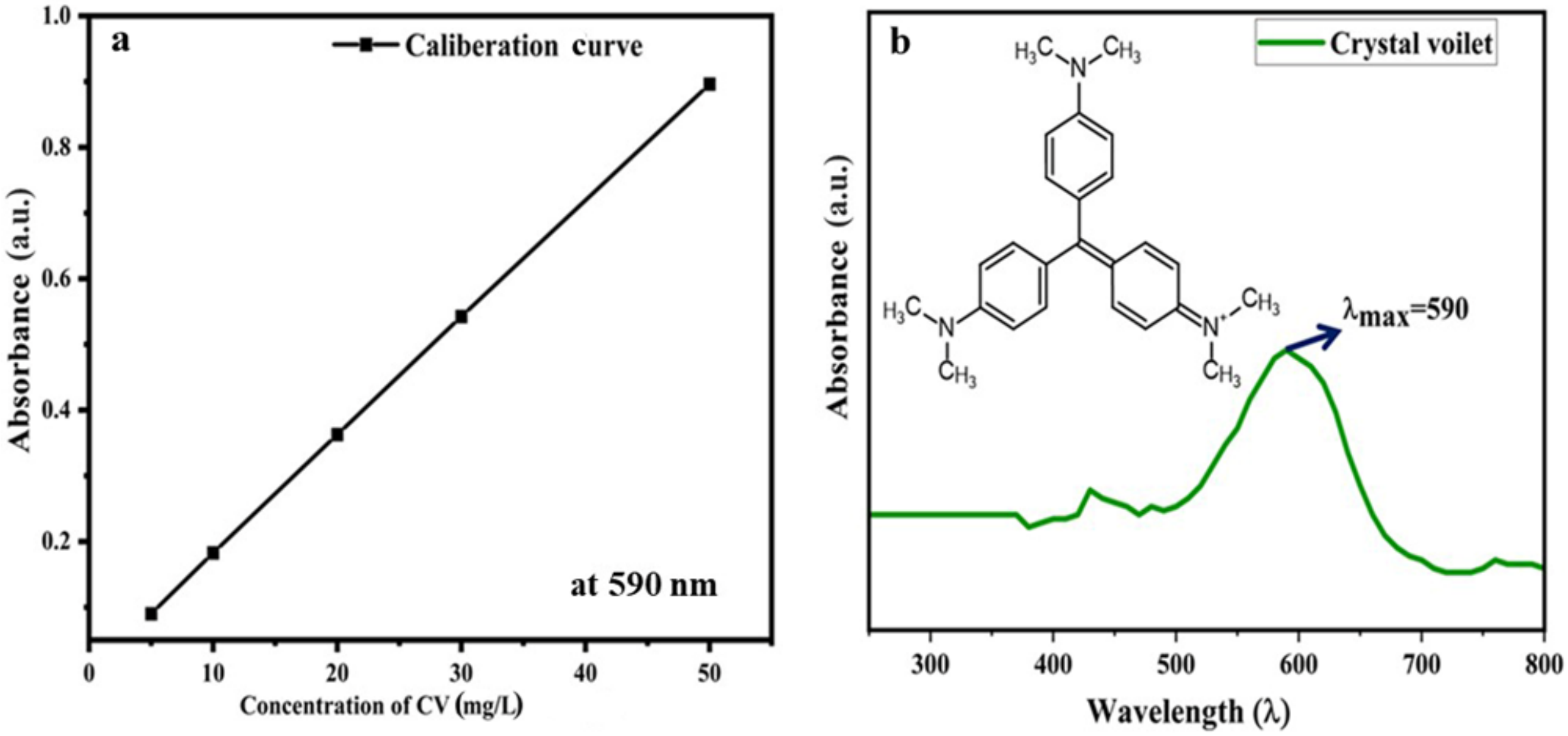


**Figure S2.** (a) Calibration curve for CV dye solution, and (b) maximum absorbance (λ max) of CV dye.

Table

**Table S1:** Comparison of representative synthesis approaches with the present phyto-mechanochemical twin-screw extrusion (TSE) strategy.

| Synthesis approach | Solvent | Processing mode and continuity | Reaction / processing time | Scalability | Energy / process-efficiency considerations | Ref. |
|---|---|---|---|---|---|---|
| Conventional wet / solution synthesis | Yes | Batch and discontinuous | Often moderate to long | Moderate. | Heating, stirring and solvent recovery can contribute substantially to process burden. | [1,2] |
| Hydrothermal / solvothermal synthesis | Yes | Batch, often under pressure and discontinuous | Typically hours to days depending on precursor, temperature and crystallization requirements. | Moderate | Requires heating of the reaction medium and pressure-resistant equipment; solvent handling remains necessary. | [3,4] |
| Conventional plant-mediated synthesis | Yes | Batch and discontinuous | Often involves extract preparation followed by reaction, washing and drying; times vary widely. | Moderate | Potentially mild reaction conditions, but extraction, solvent use and downstream drying add process steps. | [5,6] |
| Conventional ball-milling mechanochemistry | None or very low | Batch and discontinuous | Often substantially shorter than conventional solution methods, but strongly dependent on milling conditions. | Less | Reduced solvent use and direct mechanical activation are major advantages; energy input depends on mill type, speed and duration. | [7] |
| Plant-assisted mechanosynthesis | None or low; plant extract | Batch and discontinuous | Can be shorter than conventional wet synthesis; recent plant-assisted mechanochemical nanoparticle synthesis was demonstrated | Emerging; mechanochemistry offers scale-up potential, but conventional milling remains batch-oriented. | Reduced solvent demand and shorter processing can reduce process burden; specific energy must be measured for quantitative comparison. | [8] |

| | | | | | | |
|---|---|---|---|---|---|---|
| | | | under ambient conditions. | | | |
| Twin-screw extrusion mechanochemistry | None or very low | Continuous. | Short residence times are possible and can be tuned through screw speed, feed rate and screw configuration. | Strong scalability potential | Mechanochemical activation with reduced solvent demand; continuous processing can improve consistency and process efficiency. Specific energy must be experimentally quantified for a numerical energy claim. | [9,10] |
| Continuous / scalable TSE synthesis | None or low | Continuous. | Continuous residence-time control; multi-kg h−1 production demonstrated for several products. | High scalability | Mechanochemistry reduces/eliminates solvent use; continuous processing is discussed as providing consistency, smaller footprint and potential energy-efficiency benefits. | [9,10] |
| Recent review: batch vs continuous mechanochemistry | None or low | Batch and continuous-flow mechanochemical processing. | Continuous operation permits controlled residence time and process intensification. | process consistence and scale-up. | Review highlights consistency, smaller footprint and potential better energy efficiency; engineering measurements are needed for rigorous benchmarking. | [11] |
| Present phyto-mechanochemical TSE method | Low solvent | Continuous twin-screw extrusion with simultaneous feeding, mixing, shear and mechanical activation. | Use the actual optimized residence time from the manuscript; do not report a generalized time unless experimentally measured. | High potential . | Potential reduction in solvent handling and process steps. Quantitative energy efficiency was not directly measured; therefore, energy benefit should be described as potential rather than demonstrated. | Present study |

References:

1 A. Nikam, B. Prasad, A. K.- CrystEngComm and undefined 2018, *pubs.rsc.orgAV Nikam, BLV Prasad, AA KulkarniCrystEngComm, 2018•pubs.rsc.org.*

2 D. Wang, T. Xie and Y. Li, *SpringerD Wang, T Xie, Y LiNano Research, 2009•Springer*, 2009, **2**, 30–46.

3 *mdpi.comL Ndlwana, N Raleie, KM Dimpe, HF Ogutu, EO Oseghe, MM Motsa, TAM MsagatiMaterials, 2021•mdpi.com*.

4 J. Li, Q. Wu, J. W. S. and H. M. 12 and undefined 2016, *researchgate.netJ Li, Q Wu, J WuSynthesis of Nanoparticles via Solvothermal and Hydrothermal Methods 12, 2016•researchgate.net*.

5 M. Herlekar, S. Barve, R. K.-J. of Nanoparticles and undefined 2014, *Wiley Online LibraryM Herlekar, S Barve, R KumarJournal of Nanoparticles, 2014•Wiley Online Library*, 2014, **2014**, 1–9.

6 N. Rani, P. Singh, S. Kumar, P. Kumar, … V. B.-M. R. and undefined 2023, *ElsevierN Rani, P Singh, S Kumar, P Kumar, V Bhankar, K KumarMaterials Research Bulletin, 2023•Elsevier*.

7 T. Rabalao, B. Ndaba, A. Roopnarain, B. V.-R. advances and undefined 2025, *pubs.rsc.orgTM Rabalao, B Ndaba, A Roopnarain, B VatshaRSC advances, 2025•pubs.rsc.org*.

8 T. Rabalao, B. Ndaba, A. Roopnarain, B. V.-R. advances and undefined 2025, *pubs.rsc.orgTM Rabalao, B Ndaba, A Roopnarain, B VatshaRSC advances, 2025•pubs.rsc.org*.

9 R. Bolt, J. Leitch, A. Jones, … W. N.-C. S. and undefined 2022, *pubs.rsc.orgRRA Bolt, JA Leitch, AC Jones, WI Nicholson, DL BrowneChemical Society Reviews, 2022•pubs.rsc.org*.

10 D. Crawford, C. Miskimmin, A. A.-… Chemistry and undefined 2017, *pubs.rsc.orgDE Crawford, CKG Miskimmin, AB Albadarin, G Walker, SL JamesGreen Chemistry, 2017•pubs.rsc.org*.

11 R. Atapalkar, A. K.-R. C. & Engineering and undefined 2023, *pubs.rsc.orgRS Atapalkar, AA KulkarniReaction Chemistry & Engineering, 2023•pubs.rsc.org*.

**Table S2:** Twin-screw extrusion parameters for reproducible synthesis of Mt-Ag/Sn@$CeO_2$ and Ag/Sn@$CeO_2$

| **Parameter** | **Experimental condition** |
|---|---|
| Extruder | Thermo Fisher Scientific Process 11 |
| Extruder configuration | Parallel, co-rotating twin-screw |
| Screw diameter | 11 mm |
| L/D ratio | 25 |
| Screw speed | 100 rpm |
| Nominal barrel temperature | 150 °C |
| Processing atmosphere | Ambient atmosphere (air), unless otherwise specified by the instrument protocol |
| Feeding mode | Manual feeding of pre-mixed precursor/extract blend |
| Total precursor composition | 0.01 mol $(NH_4)_2[Ce(NO_3)_5]$; $AgNO_3$ = 3 mol% of Ce; $Sn(NO_3)_2$ = 3 mol% of Ce |
| Plant extract | *Melothria scabra* leaf extract, 10 mL |
| Initial homogenization | Manual mixing for 5 min before extrusion |
| Extrusion processing time | 10 min |
| Feed rate | introduced manually: quantitative feed rate was not independently measured. |

| | |
|---|---|
| Residence time | not independently measured: depends on feed and screw filling |
| Montmorillonite loading | 0.05 g hydrated Mt |
| Mt hydration | 0.05 g Mt soaked in 2.5 mL distilled water before incorporation |
| Post-extrusion drying | 80 °C for 3 h |
| Calcination temperature | 500 °C |
| Calcination duration | 4 h |
| Calcination atmosphere | Air/ambient atmosphere |
| Calcination heating rate | 5 °C min⁻$^{1}$ |
| Post-calcination processing | Cool to room temperature and gently grind/pulverize before characterization |
