# Supplementary material for: Montmorillonite-supported Ag/Sn@CeO2via phyto-mechanochemical engineering: enhanced crystal violet adsorption and CO2 capture
Source: RSC Adv. 2026 Oct 19. Online ahead of print. doi: 10.1039/d6ra07930j (PMC13679713; doi:10.1039/d6ra07930j)
Supplement: Supplementary file 1 [file RA-OLF-D6RA07930J-s001.pdf]

Supplementary Information (SI) for RSC Advances.
This journal is © The Royal Society of Chemistry

Supplementary Materials

Montmorillonite-supported Ag/Sn@CeO₂ via phyto-mechanochemical engineering: enhanced crystal violet adsorption and CO₂ capture

1.1 *Melothria Scabra* leaf extract

To prepare the extract from *Melothria scabra* leaves, fresh leaves were sourced from a local farm. They were thoroughly washed with tap water, followed by distilled water to eliminate dust and surface contaminants. After air-drying in the shade at room temperature for 3 days, the leaves were ground into a fine paste using a pestle and mortar. In a beaker, 100 mL of distilled water was mixed with 30 g of leaf paste, and the mixture was magnetically stirred at 70 °C for 1 hour. A color change was observed from green to brownish-yellow. The extract was then allowed to cool down, filtered using Whatman filter paper 01, and stored at 4°C for further use. The UV-visible spectrum of *Melothria scabra* leaf extract is shown in Fig. S1.

Figures

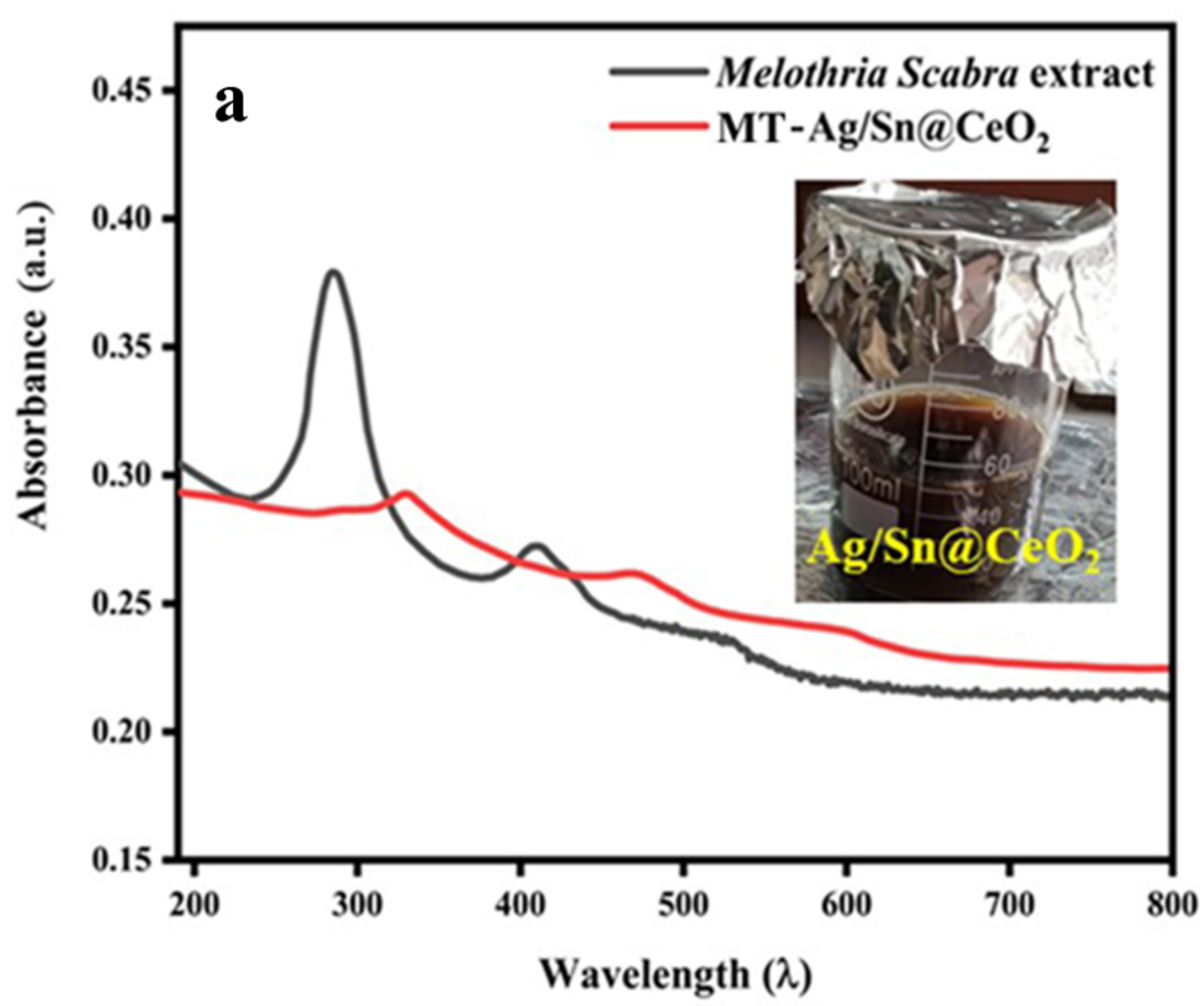

Figure S1. UV-visible spectra of *Melothria Scabra extract* and MT-Ag/Sn@CeO_2

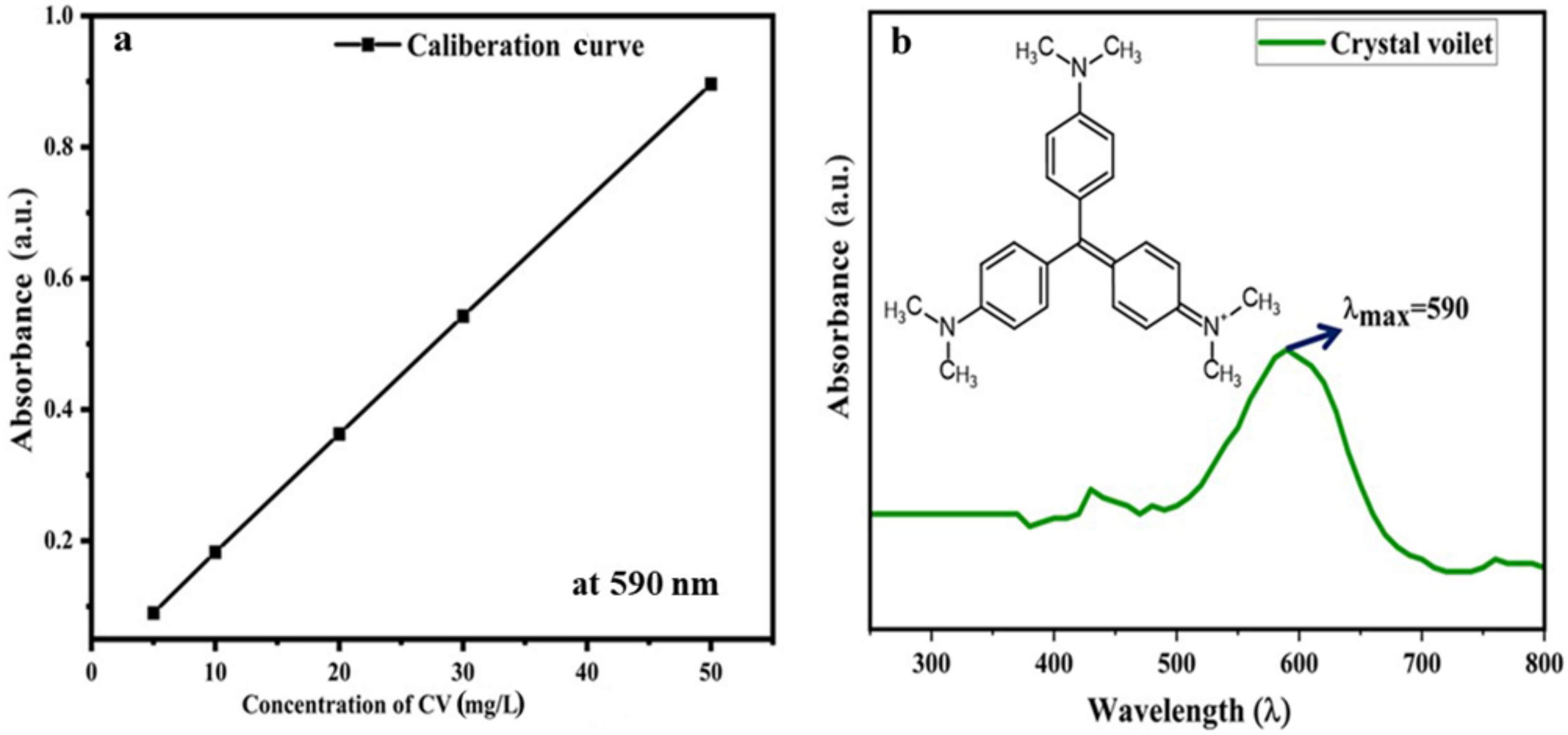

Figure S2. (a) Calibration curve for CV dye solution, and (b) maximum absorbance (λ $_{max}$) of CV dye.

Table

Table S1: Comparison of representative synthesis approaches with the present phyto-mechanochemical twin-screw extrusion (TSE) strategy.

Synthesis approach	Solvent	Processing mode and continuity	Reaction / processing time	Scalability	Energy / process-efficiency considerations	Ref.
Conventional wet / solution synthesis	Yes	Batch and discontinuous	Often moderate to long	Moderate.	Heating, stirring and solvent recovery can contribute substantially to process burden.	[1,2]
Hydrothermal / solvothermal synthesis	Yes	Batch, often under pressure and discontinuous	Typically hours to days depending on precursor, temperature and crystallization requirements.	Moderate	Requires heating of the reaction medium and pressure-resistant equipment; solvent handling remains necessary.	[3,4]
Conventional plant-mediated synthesis	Yes	Batch and discontinuous	Often involves extract preparation followed by reaction, washing and drying; times vary widely.	Moderate	Potentially mild reaction conditions, but extraction, solvent use and downstream drying add process steps.	[5,6]
Conventional ball-milling mechanochemistry	None or very low	Batch and discontinuous	Often substantially shorter than conventional solution methods, but strongly dependent on milling conditions.	Less	Reduced solvent use and direct mechanical activation are major advantages; energy input depends on mill type, speed and duration.	[7]
Plant-assisted mechanosynthesis	None or low; plant extract	Batch and discontinuous	Can be shorter than conventional wet synthesis; recent plant-assisted mechanochemical nanoparticle synthesis was demonstrated	Emerging; mechanochemistry offers scale-up potential, but conventional milling remains batch-oriented.	Reduced solvent demand and shorter processing can reduce process burden; specific energy must be measured for quantitative comparison.	[8]

			under ambient conditions.			
Twin-screw extrusion mechanochemistry	None or very low	Continuous.	Short residence times are possible and can be tuned through screw speed, feed rate and screw configuration.	Strong scalability potential	Mechanochemical activation with reduced solvent demand; continuous processing can improve consistency and process efficiency. Specific energy must be experimentally quantified for a numerical energy claim.	[9,10]
Continuous / scalable TSE synthesis	None or low	Continuous.	Continuous residence-time control; multi-kg h−1 production demonstrated for several products.	High scalability	Mechanochemistry reduces/eliminates solvent use; continuous processing is discussed as providing consistency, smaller footprint and potential energy-efficiency benefits.	[9,10]
Recent review: batch vs continuous mechanochemistry	None or low	Batch and continuous-flow mechanochemical processing.	Continuous operation permits controlled residence time and process intensification.	process consistence and scale-up.	Review highlights consistency, smaller footprint and potential better energy efficiency; engineering measurements are needed for rigorous benchmarking.	[11]
Present phyto-mechanochemical TSE method	Low solvent	Continuous twin-screw extrusion with simultaneous feeding, mixing, shear and mechanical activation.	Use the actual optimized residence time from the manuscript; do not report a generalized time unless experimentally measured.	High potential .	Potential reduction in solvent handling and process steps. Quantitative energy efficiency was not directly measured; therefore, energy benefit should be described as potential rather than demonstrated.	Present study

References:

1 A. Nikam, B. Prasad, A. K.- CrystEngComm and undefined 2018, *pubs.rsc.orgAV Nikam, BLV Prasad, AA KulkarniCrystEngComm, 2018•pubs.rsc.org.*

2 D. Wang, T. Xie and Y. Li, *SpringerD Wang, T Xie, Y LiNano Research, 2009•Springer*, 2009, **2**, 30–46.

3 *mdpi.comL Ndlwana, N Raleie, KM Dimpe, HF Ogutu, EO Oseghe, MM Motsa, TAM MsagatiMaterials, 2021•mdpi.com*.

4 J. Li, Q. Wu, J. W. S. and H. M. 12 and undefined 2016, *researchgate.netJ Li, Q Wu, J WuSynthesis of Nanoparticles via Solvothermal and Hydrothermal Methods 12, 2016•researchgate.net*.

5 M. Herlekar, S. Barve, R. K.-J. of Nanoparticles and undefined 2014, *Wiley Online LibraryM Herlekar, S Barve, R KumarJournal of Nanoparticles, 2014•Wiley Online Library*, 2014, **2014**, 1–9.

6 N. Rani, P. Singh, S. Kumar, P. Kumar, ... V. B.-M. R. and undefined 2023, *ElsevierN Rani, P Singh, S Kumar, P Kumar, V Bhankar, K KumarMaterials Research Bulletin, 2023•Elsevier*.

7 T. Rabalao, B. Ndaba, A. Roopnarain, B. V.-R. advances and undefined 2025, *pubs.rsc.orgTM Rabalao, B Ndaba, A Roopnarain, B VatshaRSC advances, 2025•pubs.rsc.org*.

8 T. Rabalao, B. Ndaba, A. Roopnarain, B. V.-R. advances and undefined 2025, *pubs.rsc.orgTM Rabalao, B Ndaba, A Roopnarain, B VatshaRSC advances, 2025•pubs.rsc.org*.

9 R. Bolt, J. Leitch, A. Jones, ... W. N.-C. S. and undefined 2022, *pubs.rsc.orgRRA Bolt, JA Leitch, AC Jones, WI Nicholson, DL BrowneChemical Society Reviews, 2022•pubs.rsc.org*.

10 D. Crawford, C. Miskimmin, A. A.-... Chemistry and undefined 2017, *pubs.rsc.orgDE Crawford, CKG Miskimmin, AB Albadarin, G Walker, SL JamesGreen Chemistry, 2017•pubs.rsc.org*.

11 R. Atapalkar, A. K.-R. C. & Engineering and undefined 2023, *pubs.rsc.orgRS Atapalkar, AA KulkarniReaction Chemistry & Engineering, 2023•pubs.rsc.org*.

Table S2: Twin-screw extrusion parameters for reproducible synthesis of Mt-Ag/Sn@CeO₂ and Ag/Sn@CeO₂

**Parameter**	**Experimental condition**
Extruder	Thermo Fisher Scientific Process 11
Extruder configuration	Parallel, co-rotating twin-screw
Screw diameter	11 mm
L/D ratio	25
Screw speed	100 rpm
Nominal barrel temperature	150 °C
Processing atmosphere	Ambient atmosphere (air), unless otherwise specified by the instrument protocol
Feeding mode	Manual feeding of pre-mixed precursor/extract blend
Total precursor composition	0.01 mol $(NH_4)_2[Ce(NO_3)_5]$; $AgNO_3$ = 3 mol% of Ce; $Sn(NO_3)_2$ = 3 mol% of Ce
Plant extract	*Melothria scabra* leaf extract, 10 mL
Initial homogenization	Manual mixing for 5 min before extrusion
Extrusion processing time	10 min
Feed rate	introduced manually: quantitative feed rate was not independently measured.

Residence time	not independently measured: depends on feed and screw filling
Montmorillonite loading	0.05 g hydrated Mt
Mt hydration	0.05 g Mt soaked in 2.5 mL distilled water before incorporation
Post-extrusion drying	80 °C for 3 h
Calcination temperature	500 °C
Calcination duration	4 h
Calcination atmosphere	Air/ambient atmosphere
Calcination heating rate	5 °C min^{-1}
Post-calcination processing	Cool to room temperature and gently grind/pulverize before characterization
